# Synthesis of Chromium(IV) Nitrides Through High-Spin Tetrahedral Chromium(I) Intermediates

**DOI:** 10.1021/acs.inorgchem.5c05944

**Published:** 2026-02-19

**Authors:** Grace B. Panetti, Matthew V. Pecoraro, Runzi Li, Junho Kim, Gabriele Hierlmeier, Paul J. Chirik

**Affiliations:** Department of Chemistry, 6740 Princeton University, Princeton, New Jersey 08544, United States

## Abstract

Reduction of (depe)_2_CrCl_2_ (depe = 1,2-bis­(diethylphosphino)­ethane) and (dep-benz)_2_CrCl_2_ (dep-benz = 1,2-bis­(diethylphosphino)­benzene) under 1 atm of N_2_ furnished the dinitrogen complexes (depe)_2_Cr­(N_2_)_2_ and (dep-benz)_2_Cr­(N_2_)_2_, respectively. One-electron oxidation of these products with FcBAr^F^_4_ (Fc = ferrocenium, BAr^F^_4_ = B­(3,5-(CF_3_)_2_C_6_H_3_)_4_) yielded the unusual, high-spin tetrahedral complexes [(depe)_2_Cr]­[BAr^F^_4_] and [(dep-benz)_2_Cr]­[BAr^F^_4_] with concomitant loss of dinitrogen. Reaction of the chromium­(I) derivatives with Ph_3_CN_3_ furnished rare examples of chromium­(IV) nitrides as confirmed spectroscopically and by X-ray crystallography. While [(depe)_2_Cr(≡N)][BAr^F^_4_] underwent association of isocyanides accompanied by partial ligand dissociation, neither chromium nitride was reactive toward H_2_ or diphenylsilane under thermal or photochemical conditions. These results distinguish the unique properties of the chromium­(IV) nitrides as compared to heavier group 6 congeners and demonstrate both the feasibility of nitride synthesis and the limitations of dinitrogen cleavage and subsequent N–H bond formation.

## Graphical abstract

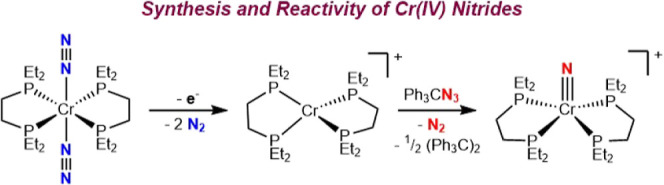

## Introduction

The catalytic conversion of dinitrogen to ammonia with molecular compounds is a long-standing and formidable challenge in coordination chemistry. While the Haber-Bosch process has enabled industrial-scale fertilizer production for over a century,^1^ its high energy input and reliance on pressurized hydrogen motivate the search for molecular examples capable of N_2_ reduction under milder conditions.^2^ Such systems offer insight into the fundamental steps of N≡N bond cleavage^3^ and N–H bond formation,^4^ with the goal of ultimately enabling more sustainable ammonia synthesis.

The coordination, subsequent activation and functionalization of dinitrogen with coordination and organometallic complexes is well-precedented across the transition metals^5^ and examples of ammonia synthesis using the addition of protons and reductants has been extensively examined.^6^ Among these, molybdenum and tungsten complexes have been among the most prominent, tracing to the foundational studies of Chatt^7^ and later expanded by Hidai,^8^ who demonstrated that bis­(phosphine) supported molybdenum and tungsten complexes coordinate dinitrogen and generate ammonia upon treatment with strong acids, typically forming intractable metal-containing byproducts.^9^

Our laboratory has been exploring approaches to ammonia synthesis from early transition metal dinitrogen and nitride complexes using H_2_ as the reductant and source of hydrogen atoms (Scheme 1a).^10–12^ Motivation for these studies is understanding how N–H bonds form using H_2_ and the thermodynamics associated with these transformations with the goal of ammonia synthesis with minimal chemical overpotential. An initial demonstration of these concepts was the hydrogenation of the N_2_-derived molybdenum nitride, [(depe)_2_Mo≡N]­[BAr^F^_4_] upon irradiation with visible light using (η^5^-C_5_Me_5_)­Ir­(ppy)H (ppy = 2-phenylpyridine) as the photocatalyst.^10^ Ammonia was formed in 38% yield, with catalyst deactivation arising from C−H reductive elimination of phenylpyridine.

Control experiments established that (η^5^-C_5_Me_5_)­Ir­(ppy)H served as a photocatalyst and that the iridium-hydride did not participate in N–H bond formation. Use of the more photostable *fac*-Ir­(ppy)_3_ enabled near quantitative formation of ammonia and demonstration of a synthetic cycle where the molybdenum pentahydride product was converted back to the starting metal nitride through N_2_ cleavage.^11^ Complementary studies with tungsten demonstrated that one-electron oxidation of *trans*-(depe)_2_W­(N_2_)_2_ resulted in dinitrogen scission through an observed bimetallic intermediate to form the corresponding nitride, [(depe)_2_W≡N]­[BAr^F^_4_].^12^ Photodriven hydrogenation in the presence of *fac*-Ir­(ppy)_3_ and irradiation with blue LEDs released free NH_3_ albeit in modest yield, demonstrating that this approach is effective for N–H bond formation and ultimately breaking strong metal-nitrogen bonds.

**Scheme 1:**
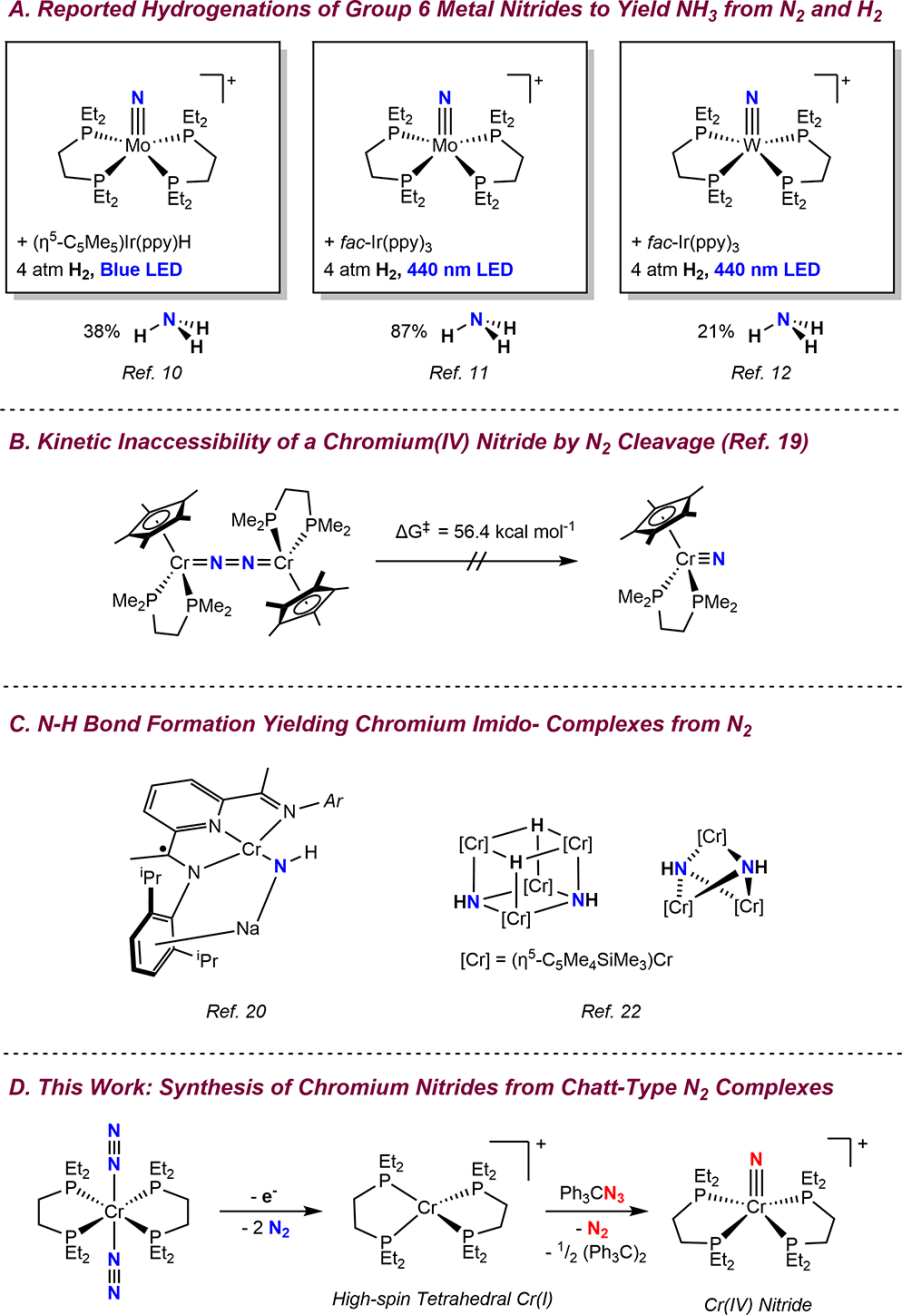
Examples of Chromium, Molybdenum, and Tungsten Nitrides Relevant to Dinitrogen Activation; (A) Reported Hydrogenations of Group 6 Metal Nitrides to Yield NH_3_ from N_2_ and H_2_; (B) Kinetic Inaccessibility of a Chromium­(IV) Nitride by N_2_ Cleavage; (C) N–H Bond Formation Yielding Chromium Imido-Compounds from N_2_; (D) This Work: Synthesis of Chromium Nitrides from Chatt-Type N_2_ Complexes

Despite these advances, the analogous chemistry with chromium for N_2_ cleavage, nitride formation and ammonia synthesis is underexplored.^13,14^ Notably, both Cr­(I) and Cr­(III) oxidation states are accessible and needed to enable formation of a 10 π-electron M=N=N=M bridge in 4- and 3-fold symmetric ligand fields, respectively.^15^ These electronic configurations suggest the potential for analogous pathways to molybdenum and tungsten for N_2_ cleavage and motivate deeper exploration of chromium-based nitrogen-fixation chemistry. These variable oxidation states, accessible redox potentials, and capacity to support multiple bonding modes make chromium a potentially versatile metal for small-molecule activation yet few such compounds have been explored.

The limited exploration of chromium in nitrogen fixation chemistry can be attributed in part to the scarcity of well-characterized chromium–dinitrogen complexes. This deficiency has been attributed to weak to minimal π-backbonding that renders N_2_ coordination unfavorable.^16^ When formed, these complexes are often labile with N_2_ ligands that readily dissociate in the absence of strongly donating, chelating ligands.^13,17^ As such, terminal chromium–dinitrogen complexes remain underrepresented though the number of reported examples continues to increase.^14^

Recent reports of bridging dinitrogen complexes of chromium have begun to expand the scope of its coordination chemistry and suggest potential for N_2_ activation and cleavage.^18–20^ These compounds, often stabilized by multidentate ligands, demonstrate that chromium supports end-on coordination in multimetallic structures. Despite these advances, cleavage of N_2_ to form a terminal chromium nitride has not yet been demonstrated.

Most known chromium nitrides have been synthesized by nitride transfer reagents and are typically Cr­(VI), limiting relevance to nitrogen fixation.^21^ The paucity of N_2_-derived chromium nitrides reflects both kinetic and thermodynamic challenges associated with N≡N bond scission with first-row transition metals. Theopold and co-workers recently reported the synthesis and characterization of dinitrogen and nitride complexes of [(η^5^-C_5_Me_5_)­Cr­(dmpe)], establishing prohibitively high barriers to N≡N bond cleavage and nitride coupling that preclude interconversion under thermally accessible conditions (Scheme 1b).^19^ Independently, Budzelaar and co-workers reported a pyridine­(diimine)-supported chromium dinitrogen complex that promotes reductive functionalization and N–H bond formation upon treatment with sodium hydride to afford a sodium-chromium bridging imido compound.^20^ In complementary studies, Luo, Hou and co-workers reported that cyclopentadienyl-supported di- and trinuclear chromium hydrides derived from H_2_ are capable of both cleaving N_2_ and forming N–H bonds, yielding multinuclear compounds featuring bridging imide and nitride ligands (Scheme 1c).^22^ These findings collectively underscore the potential of chromium to access reactive nitrido and imido products under the appropriate electronic and geometric conditions, motivating further exploration of its role in molecular nitrogen fixation. Protonation of chromium–dinitrogen complexes has been shown to promote N–H bond formation with strong acids,^23,24^ and silylation of chromium complexes with various nitrogen-containing ligands has also been demonstrated.^23d,25^

Here we describe the synthesis, characterization and reactivity of a rare example of a chromium­(IV) nitride, [(depe)_2_Cr­(N)]­[BAr^F^_4_], the structural analog of the molybdenum and tungsten congeners (Scheme 1d). One-electron oxidation of the chromium(0) dinitrogen compound resulted in N_2_ dissociation to form the unusual, tetrahedral cationic chromium­(I) derivative, [(depe)_2_Cr]­[BAr^F^_4_], where addition of triphenylmethyl azide resulted in expulsion of N_2_ and formation of the chromium­(IV) nitride. Attempts to hydrogenate the chromium nitride under conditions that are effective for its molybdenum and tungsten counterparts were unsuccessful, highlighting a divergence in reactivity. These findings complete a systematic structural and reactivity comparison among the group 6 triad and provides insights into how the identity of the metal modulates the key steps of dinitrogen activation and photodriven hydrogenation to ammonia.

## Results and Discussion

### Synthesis of Cationic Cr­(IV) Nitrides from Bis­(phosphine)-Supported Cr(0) Dinitrogen Compounds

Our investigations commenced with the synthesis of the previously reported compound (depe)_2_Cr­(N_2_)_2_ (**Cr1**),^24^ with the goal of targeting one-electron oxidation as a strategy to promote N≡N bond cleavage through formation of a 10 π-electron M=N=N=M intermediate from Cr­(I).^15^ This approach was successfully applied by Masuda^26^ and later our laboratory^12^ for the synthesis of the molybdenum and tungsten congeners, respectively. Consistent with the reported literature procedure, **Cr1** was prepared by the reduction of (depe)_2_CrCl_2_ with excess Mg^0^ in THF with ^1^H and ^31^P NMR and IR spectroscopic data in agreement with the previous report (Scheme 2).^24^

**Scheme 2:**
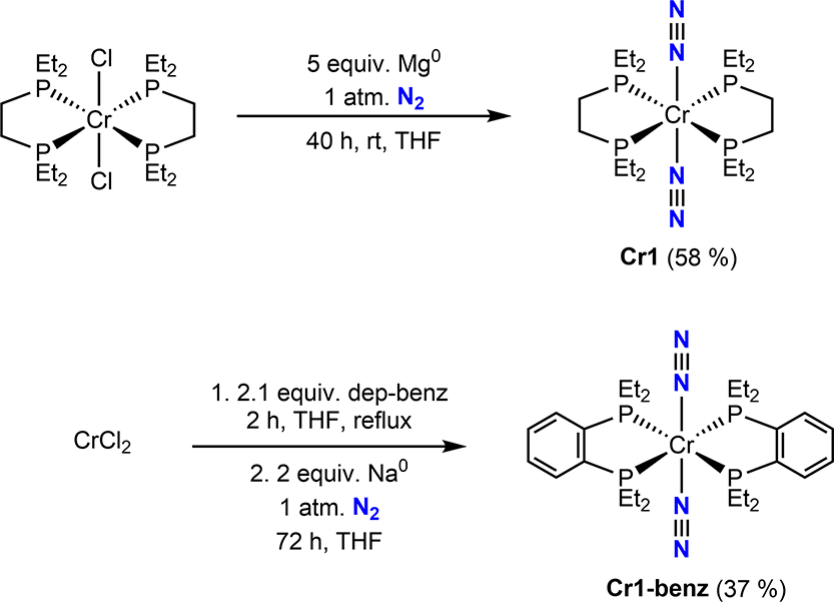
Synthesis of chromium(0) Dinitrogen Complexes

A modification of this procedure was also applied to a benzannulated analog of **Cr1**, (dep-benz)_2_Cr­(N_2_)_2_ (**Cr1**-**benz**) where reduction of in situ-generated (dep-benz)_2_CrCl_2_ with Na metal furnished the desired product as a red solid in 37% yield (Scheme 1). The rigid arene-linked backbone was included to suppress ligand dissociation commonly observed with first-row transition metal complexes, including chromium.^27^ The benzene-*d*_6_^31^P­{^1^H} NMR spectrum of **Cr1-benz** exhibited a single resonance at 78.4 ppm, similar to that of **Cr1** at 80.1 ppm. A strong ν­(N_2_) band at 1930 cm^–1^ was observed in the solid-state (KBr) infrared spectrum, consistent with the terminal dinitrogen stretch of 1917 cm^–1^ reported for **Cr1**.

Single crystals of **Cr1-benz** were obtained from a concentrated toluene solution stored at −35 °C. The solid-state structure confirmed the assignment as the chromium(0) complex with *trans* dinitrogen ligands and each of the benzo rings splayed in an anti arrangement (Figure 1). The Cr–N and N–N bond lengths of 1.8919(11) and 1.1162(16) Å, respectively, are indistinguishable from those reported for **Cr1**.^16^ These metrics are consistent with minimal backbonding and activation of the N_2_ ligand, especially when compared to the 1.10 Å N≡N distance in free dinitrogen.^28^

**Figure 1:**
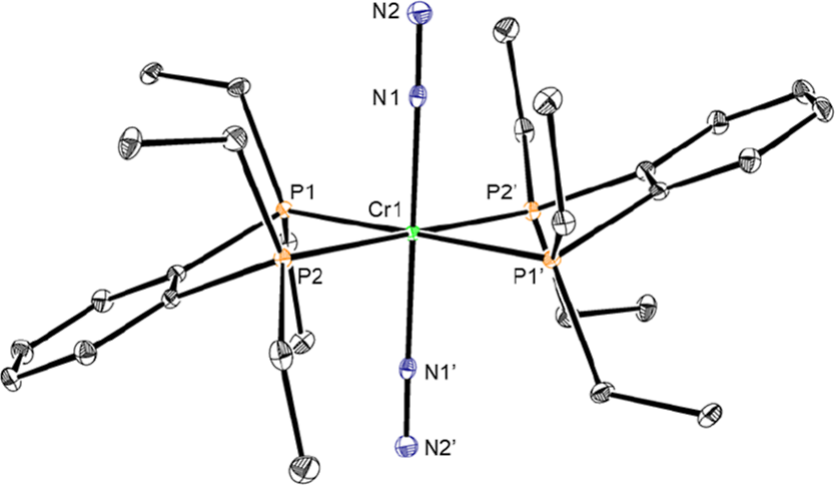
Representation of the solid-state structure of **Cr1-benz** with 30% probability ellipsoids. Hydrogen atoms omitted for clarity.

With both **Cr1** and **Cr1-benz** in hand, their one-electron oxidation reactivity was explored under a dinitrogen atmosphere. Notably, Mock and co-workers previously reported that **Cr1** exhibits reversible one-electron oxidation by cyclic voltammetry, suggesting the one-electron oxidized compound is observable at appropriate scan rates,^16^ similar to results reported by Masuda on the analogous molybdenum complex.^26^

Treatment of a diethyl ether solution of **Cr1** with one equivalent of FcBAr^F^_4_ resulted in an immediate color change from red to yellow with concomitant evolution of N_2_ gas. The THF-*d*_8_^1^H NMR spectrum of the unpurified reaction mixture only exhibited signals for the BAr^F^_4_ anion and free ferrocene. The ^31^P­{^1^H} NMR spectrum confirmed complete consumption of **Cr1** with no new detectable resonances, consistent with formation of a paramagnetic product. Analysis by infrared and Raman spectroscopies provided no evidence for the presence of terminal or bridging N_2_ ligands, consistent with formation of the cationic chromium­(I) derivative, [(depe)_2_Cr]­[BAr^F^_4_] (Scheme 3, **Cr2**).

**Scheme 3:**
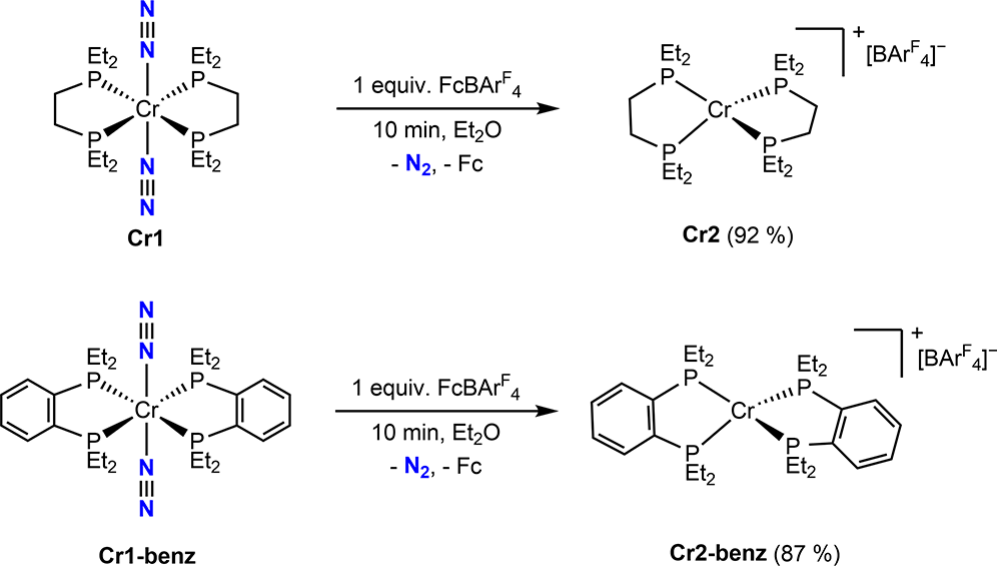
Synthesis of bis­(phosphine) chromium­(I) Compounds by One-Electron Oxidation

Although attempts to isolate diffraction quality crystals of **Cr2** with the BAr^F^_4_ counterion were unsuccessful, repeating the synthesis with Fc*BAr^F^_20_ (Fc* = decamethyl ferrocenium; BAr^F^_20_ = B­(C_6_F_5_)_4_) and layering a THF solution of the crude product with pentane at −35 °C provided single crystals suitable for X-ray diffraction of the corresponding BAr^F^_20_ complex (**Cr2′**). The solid-state structure of **Cr2′** (Figure 2) confirms formation of an idealized tetrahedral chromium­(I) complex (τ_4_ = 0.67).^29^ The syntheses of **Cr2** and **Cr2′** are rare examples of bis­(phosphine) supported chromium­(I) compounds.^30^ Likewise, oxidation of **Cr1-benz** with FcBAr^F^_24_ in diethyl ether followed by recrystallization from a THF solution layered with pentane furnished orange crystals suitable for X-ray diffraction that confirmed formation of **Cr2-benz**. As with **Cr2′**, an idealized tetrahedral cationic chromium­(I) compound (τ_4_ = 0.73) was observed.

**Figure 2:**
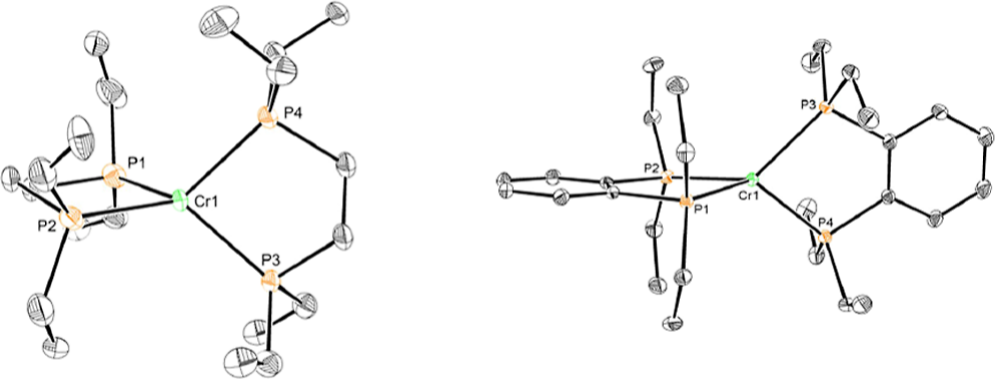
Representations of the solid-state structures of **Cr2′** (left) and **Cr2-benz** (right) at 30% probability ellipsoids. Hydrogen atoms and counterions (BAr^F^_20_ and BAr^F^_4_, respectively) omitted for clarity.

X-Band EPR spectra of **Cr2, Cr2′** and **Cr2-benz** were collected in 2-MeTHF at 25 K and are consistent with *S* = ^5^/_2_ chromium compounds with pronounced zero-field splitting (ZFS) (Figure 3). For **Cr2** and **Cr2′**, resonances spanning approximately 6–660 mT were observed, while **Cr2-benz** displayed signals from approximately 10 to 660 mT. No discernible differences were detected between spectra obtained for the depe supported cation generated by oxidation with FcBAr^F^_4_ **(Cr2)** or Fc*BAr^F^_20_ (**Cr2′**) indicating little effect of the supporting anion. Simulation of the EPR spectra for **Cr2** and **Cr2′** provided a *g* value of 1.943, a ZFS parameter *D* = 3495.8 MHz, and an *E*/*D* ratio of 0.030. In contrast, **Cr2-benz** was best modeled using *g* = 2.001, *D* = 3798.46 MHz, and *E*/*D* = 0.042, signaling increasing rhombicity. The observation of a rhombic signal is notable given the approximately tetrahedral coordination geometry observed in the solid state and may reflect geometric distortion in solution and the frozen glass.

**Figure 3:**
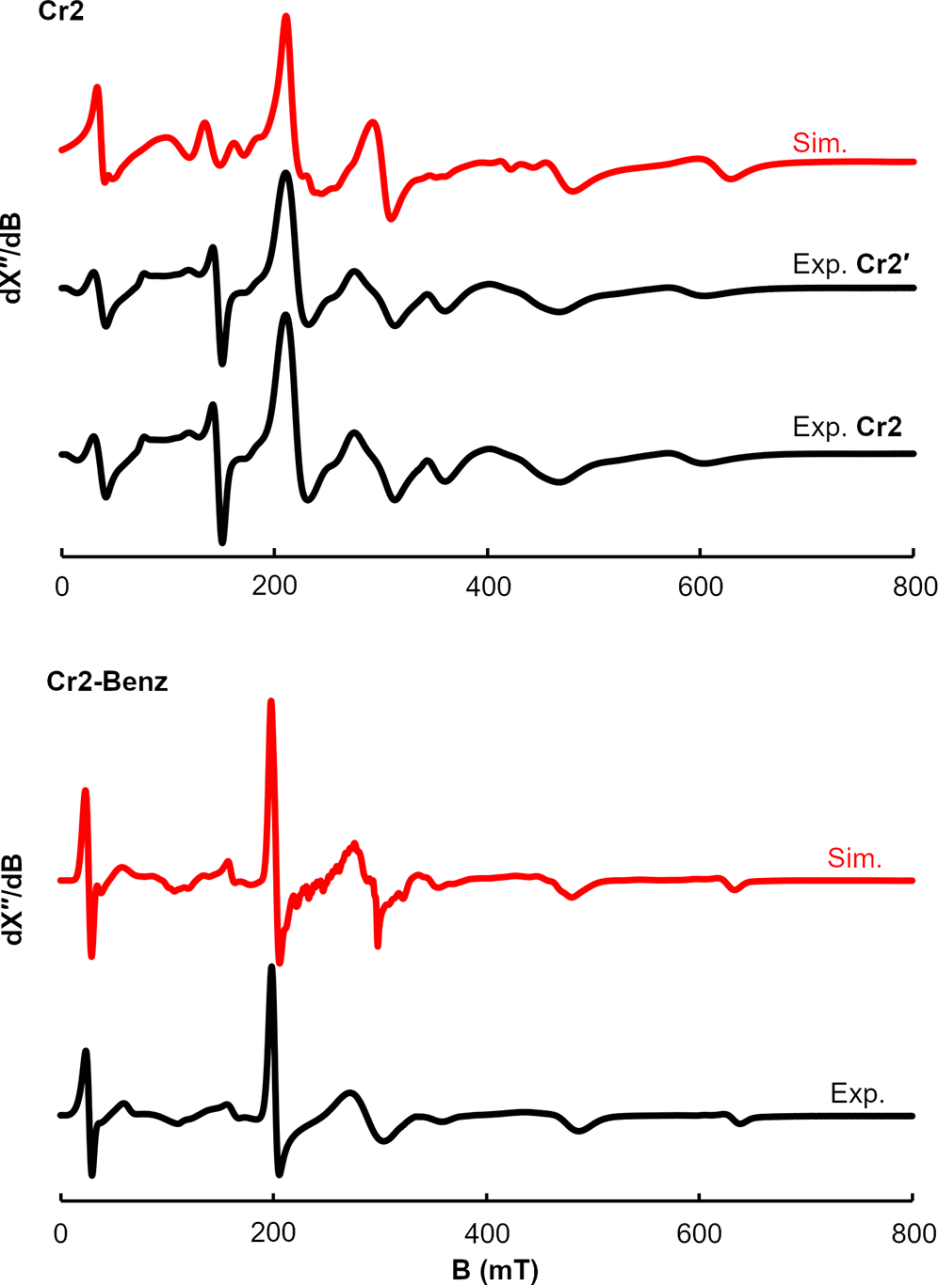
X-Band EPR spectra collected at 25 K in 2 Me-THF glass. Experimental parameters: microwave frequency = 9.365 GHz, power = 2.000 mW, and modulation amplitude = 4.000 G. Simulation parameters: **2**: *S* = 5/2, *g* = 1.943, *D* = 3495.8, *E*/*D* = 0.030, *D*_strain_ = (103.34, 2149.14); **2-Benz** *S* = 5/2, *g* = 2.001, *D* = 3798.46, *E*/*D* = 0.042, *D*_strain_ = (134.00, 226.92).

The loss of dinitrogen upon oxidation of the chromium(0) dinitrogen compounds prompted evaluation of the reactivity of the chromium­(I) derivatives with N-atom transfer reagents. Triphenylmethyl azide (**Ph**_**3**_ **CN**_**3**_) has been previously targeted as an N-atom source in the synthesis of metal nitrides; however the reported examples have been limited to reactions that result in metal imido- and azide complexes.^31^ Treating a THF solution containing either **Cr2** or **Cr2-benz** with 1 equivalent of **Ph**_**3**_ **CN**_**3**_ resulted in a color change from yellow or orange to green along with rapid gas evolution. Analysis by ^31^P­{^1^H} NMR spectroscopy revealed the appearance of new diagnostic singlets at 76.18 and 78.69 ppm for **Cr3** and **Cr3-benz**, respectively, signaling formation of a diamagnetic chromium product. The ^1^H NMR resonances for the ethyl groups on the phosphine ligand were inequivalent, consistent with formation of the chromium nitride and dissymmetry above and below the metal chelate plane. Repeating the procedure with ^15^N-labeled Ph_3_CN_3_ (50% ^15^N at the α-position and ^15^N 50% at the γ-position) and analysis by ^15^N NMR spectroscopy revealed a resonance at 1026 ppm for **Cr3**, consistent with the values of 979 and 1020 ppm for Cr­(VI) nitrides reported by Cummins and co-workers.^32^ Together, the combined spectroscopic characterization is consistent with the formulation of **Cr3** and **Cr3-benz** as Cr­(IV) nitrides (Scheme 4).

**Scheme 4:**
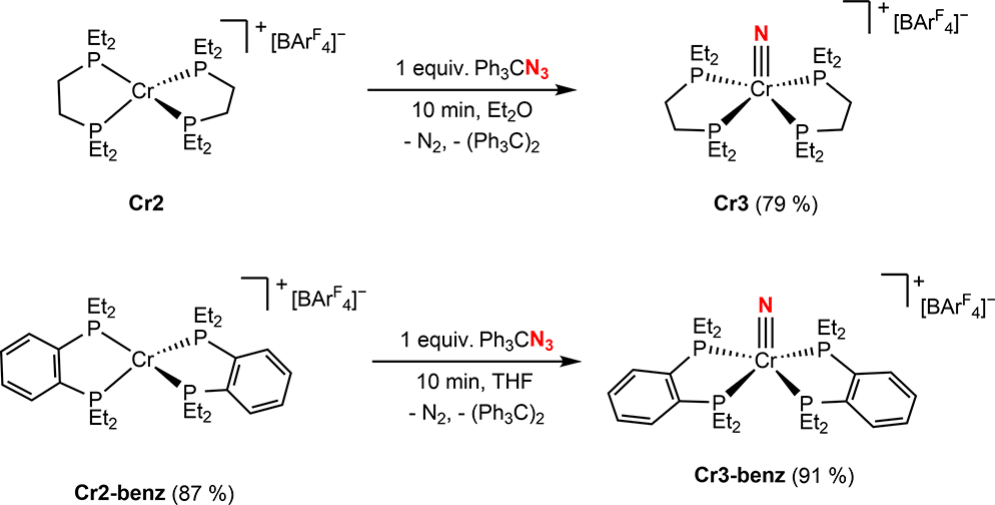
Synthesis of chromium­(IV) Nitride Compounds

Measurement of the ^15^N NMR chemical shift for **Cr3** enables comparison to other related group 6 metal nitrides supported by bidentate phosphine ligands. The chemical shift of 1026 ppm for **Cr3** is significantly downfield from the value of 851 ppm reported for the molybdenum congener^26^ as well as the shift of 836 ppm for [(dppe)_2_W­(N)]­[BAr^F^_4_] (dppe = 1,2-bis­(diphenylphosphino)­ethane).^12^ These progressive upfield shift observed upon descending the group 6 triad is likely a result of strengthened metal-nitrogen multiple bonding and increased metal-nitride covalency for the heavier metals.

Crystals of **Cr3** suitable for single-crystal X-ray diffraction were obtained by layering a diethyl ether solution of the complex with pentane at −35 °C (Figure 4). Single crystals of **Cr3-benz** were obtained from a THF/pentane mixture. The molecular structures of both complexes confirmed formation of the chromium nitrides, featuring Cr–N bond lengths of 1.543(8)–1.552(7) Å for **Cr3** and 1.540(5)–1.569(9) Å for **Cr3-benz**. By comparison, the molybdenum and tungsten analogs of **Cr3** exhibit longer metal–nitrogen bond distances, with Mo–N lengths of 1.640(5)-1.653(4) Å^26^ and a W–N bond distance of 1.675(7) Å.^12^

**Figure 4:**
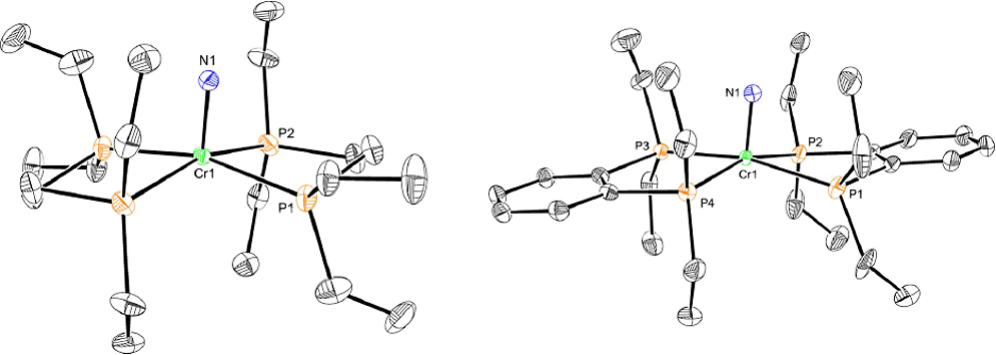
Representation of the solid-state structures of **Cr3** (left) and **Cr3-benz** (right) at 30% probability ellipsoids. Hydrogen atoms and BAr^F^_4_ counterions omitted for clarity.

Cyclic voltammetry experiments were conducted on **Cr3** and **Cr3-benz** in THF using 0.1 M tetrabutylammonium hexafluorophosphate (TBAPF_6_) as the supporting electrolyte (Figures S32 and S33). For **Cr3**, an oxidation event assigned to the Cr­(IV)/Cr­(V) couple was observed at −0.572 V vs Fc^+^/Fc and was electrochemically reversible, while a reduction event attributed to the Cr­(IV)/Cr­(III) couple occurred at −2.59 V and was quasi-reversible. In contrast, **Cr3-benz** exhibits a reversible oxidation at −0.255 V and a reversible reduction at −2.42 V vs Fc^+^/Fc. Comparison of the redox potentials indicates that oxidation is more accessible for the depe-supported complex, whereas reduction is more accessible for the dep-benz analog, consistent with increased electron donation from the depe ligand relative to dep-benz.

### Reactivity of the Cr­(IV) Nitrides

The reactivity of the isolated Cr­(IV) nitrides was investigated with isonitriles, diphenylsilane, and H_2_. Previous studies with [(depe)_2_Mo­(N)]­[BAr^F^_4_] established that excess phosphine inhibits photodriven hydrogenation, and that the benzannulated analog, [(dep-benz)_2_Mo­(N)]­[BAr^F^_4_], was less reactive under identical conditions, suggesting that phosphine dissociation may occur during hydrogenation.^11^ Motivated by this precedent, reaction of the chromium examples with a neutral ligand was examined to determine whether coordination would occur *trans* to the nitride and whether rearrangement or no reactivity would be observed when using more coordinating substrates than H_2_. To evaluate this possibility, 2,6-dimethylphenylisocyanide was selected as a representative substrate to avoid potential steric interactions with the ethyl substituents of the chelating phosphines.

Addition of one equivalent of 2,6-dimethylisocyanide to a diethyl ether solution of **Cr3** resulted in an immediate color change from green to red (Scheme 5). The THF-*d*_8_^31^P­{^1^H} NMR spectrum of **Cr4** is consistent with a square-pyramidal chromium­(IV) nitride with an apical nitride ligand and an equatorial plane comprising one κ^2^-depe, one κ^1^-depe, and one coordinated 2,6-dimethylisocyanide. Four distinct, mutually coupled phosphorus resonances were observed: P1 at −5.52 ppm (d, *J*_P1‑P2_ = 28.2 Hz), P2 at 55.98 ppm (ddd, *J*_P1‑P2_ = 28.2 Hz, *J*_P2‑P3_ = 34.7 Hz, *J*_P2‑P4_ = 6.4 Hz), P3 at 60.89 ppm (dd, *J*_P2‑P3_ = 34.7 Hz, *J*_P3‑P4_ = 19.8 Hz), P4 at 72.13 ppm (dd, *J*_P2‑P4_ = 6.2 Hz, *J*_P3‑P4_ = 20.1 Hz). Notably, the upfield resonance at −5.52 ppm couples only to P2 and appears in the region typical of free trialkylphosphines (e.g., depe = −18.8 ppm),^33^ supporting its assignment as the “dangling” phosphorus of the κ^1^-depe ligand.^34^

**Scheme 5:**
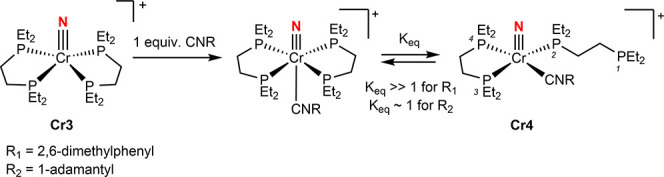
Reactivity of Cr3 with 2,6-Dimethylphenyl-isocyanide and 1-Adamantyl Isocyanide

When 1-adamantyl isocyanide was used in place of 2,6-dimethylphenylisocyanide, analogous reactivity was observed. The THF-*d*_8_^31^P­{^1^H} NMR spectrum supported the formation of two chromium-containing compounds in an approximately 1:1 ratio (Figure S23). The first exhibited chemical shifts and coupling patterns closely matching those of **Cr4** and was assigned accordingly. The second exhibits a singlet at 72.61 ppm and is assigned as (depe)_2_Cr­(≡N)­(C≡NAd), featuring an isocyanide ligand bound *trans* to the nitride. These observations are consistent with the establishment of an equilibrium between a *trans*-bound isocyanide compound and a geometrically reorganized complex resulting from partial phosphine dissociation.

Single crystals of **Cr4** suitable for X-ray diffraction formed slowly over one week at −35 °C upon vapor diffusion of pentane into a saturated solution of the complex in THF. (Figure 5). The principal difference between the observed solid state and solution structures is that the previously “dangling” phosphine arm is not free in the former but instead occupies the site *trans* to the nitride, with a long Cr–P separation of 2.7270(15) Å-substantially elongated relative to the remaining Cr–P bonds (2.3807(15), 2.3864(15), and 2.4080(15) Å) due to the *trans* influence of the nitride ligand. The observed coordination of the isocyanide indicates that coordination of L-type ligands to the nitride in cationic bis­(phosphine) chromium complexes is possible but may require concomitant phosphine dissociation and geometric rearrangement.

**Figure 5:**
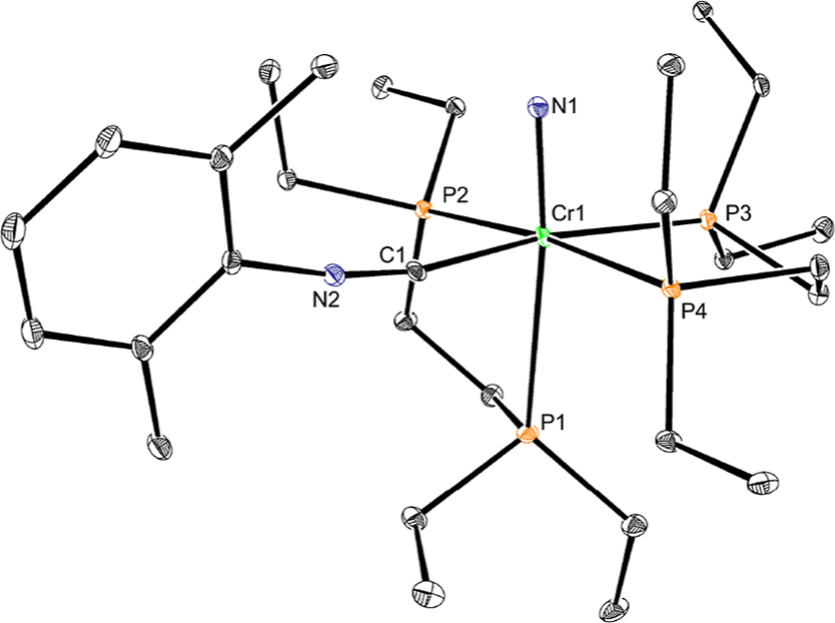
Representation of the solid-state structure of **Cr4** with 30% probability ellipsoids. Hydrogen atoms and BAr^F^_4_ counterion omitted for clarity.

The thermal and photochemical hydrogenation reactivity of **Cr3** were also examined. In contrast to its molybdenum and tungsten congeners, no reaction was observed under any of the previously reported hydrogenation conditions. Likewise, treatment of a THF solution of **Cr3** with either (η^5^-C_5_Me_5_)­Ir­(ppy)H or (η^5^-C_5_Me_5_)­Ir­(bq)­H^10^ (bq = benzo­[h]­quinolinyl) resulted in minor decomposition upon irradiation with blue LEDs. Even under the optimized photochemical conditions for the molybdenum and tungsten complexes^11,12^ —irradiation with Ir­(ppy)_3_ under 4 atm of H_2_—no ammonia formation was detected with only slight decomposition of the chromium nitride. Analogous reactions with **Cr3-benz** similarly showed a lack of productive reactivity. These observations highlight a clear divergence in reactivity within the Group 6 series, rendering chromium as distinct from its heavier congeners under identical conditions.

One possibility for the lack of reactivity may be the inability of the chromium nitride to engage in productive 1,2-addition chemistry. To evaluate this hypothesis, diphenylsilane (**Ph**_**2**_ **SiH**_**2**_) was added to a THF-*d*_8_ solution of **Cr3** and no reactivity was observed upon heating to 60 °C or irradiation with blue LEDs. In contrast, the corresponding molybdenum nitride has been shown to readily undergo Si–H 1,2-addition under analogous conditions.^35^ This absence of silane activation mirrors the lack of hydrogenation reactivity and further supports the conclusion that chromium does not readily engage in 1,2-addition pathways under conditions that are productive for its heavier congeners.

## Conclusions

The synthesis of unusual, tetrahedral, high-spin bis­(phosphine) chromium­(I) complexes from one-electron oxidation of the corresponding chromium(0) bis­(dinitrogen) complexes was accomplished. Treatment of these compounds with triphenylmethyl azide as an N-atom source enabled the synthesis and isolation of rare chromium­(IV) terminal nitride complexes. Coordination of isocyanides was observed with concomitant phosphine dissociation and geometric reorganization in solution. Attempted thermal and photochemical hydrogenation and hydrosilylation of these compounds was unsuccessful, highlighting key differences between the first-row metal and the heavier, second- and third-row congeners.

## Experimental Section

### General Considerations

All air- and moisture-sensitive manipulations were carried out using vacuum line, Schlenk and cannula techniques or in an MBraun inert atmosphere nitrogen dry box unless otherwise noted. All glassware was stored in a preheated oven (≥150 °C) prior to use. Pentane, benzene, toluene, diethyl ether, and tetrahydrofuran used for air- and moisture-sensitive manipulations were dried and deoxygenated using literature procedures.^36^ Benzene-*d*_6_ used for NMR spectroscopy was distilled from sodium metal and stored over 4 Å molecular sieves. THF-*d*_8_ used for NMR spectroscopy was dried using sodium-benzophenone ketyl^37^ and directly vacuum transferred to the reaction mixtures prior to use. Celite, alumina, and silica were dried at 180 °C under vacuum for 3 days prior to use in the glovebox. Solid reagents were dried under vacuum overnight and stored under dinitrogen prior to use. All compounds were purchased from Sigma-Aldrich, Alfa Aesar, Tokyo Chemical Industry, and Acros Organics and used as received unless otherwise stated. No uncommon hazards are noted.

^1^H NMR spectra were recorded on either Bruker AVANCE 300, 400 or 500 spectrophotometers operating at 300.13 MHz, 399.8 and 500.46 MHz, respectively. ^13^C NMR spectra were recorded on either Bruker Avance 300, 400 or 500 spectrometers operating at 75.48 MHz, 100.54 and 125.85 MHz, respectively. All ^1^H and ^13^C NMR chemical shifts are reported in ppm relative to SiMe_4_ using the ^1^H and ^13^C chemical shifts of the solvent^38^ as a standard. ^1^H NMR data for diamagnetic compounds are reported as follows: chemical shift, multiplicity (s = singlet, d = doublet, t = triplet, q = quartet, p = pentet, br = broad, m = multiplet, app = apparent, obsc = obscured), coupling constants (Hz), integration, assignment. ^13^C NMR data for diamagnetic compounds are reported as follows: chemical shift, number of protons attached to carbon (e.g., CH_2_), assignment. ^2^H NMR spectra were recorded on Bruker Avance 400 or 500 spectrometers operating at 61.42 and 76.88 MHz, respectively and referenced to TMS-*d*_12_ as an external standard. ^19^F NMR spectra were recorded on Bruker Avance 400 or 500 spectrometers operating at 376.19 MHz and 470.96 MHZ, respectively, and referenced to CFCl_3_ as an external standard. ^31^P NMR spectra were recorded on either Bruker Avance 400 or 500 spectrometers operating at 161.84 and 202.00 MHz, respectively, and were referenced to 85% H_3_PO_4_ as an external standard. ^15^N NMR spectra were recorded on a Bruker Avance 400 spectrometer operating at 40.51 MHz and referenced to ^15^NH_3_ as an external standard.

Continuous wave EPR spectra were recorded at room temperature or 20 K on an X-band Bruker EMXPlus spectrometer equipped with an EMX standard resonator and a Bruker PremiumX microwave bridge. The spectra were simulated using EasySpin for MATLAB.^39^ Elemental analyses were performed at Robertson Microlit Laboratories, Inc., in Ledgewood, NJ. Infrared spectroscopy was conducted on a Thermo-Nicolet iS10 FT-IR spectrometer calibrated with a polystyrene standard.

Cyclic voltammetry measurements were performed under an inert atmosphere of nitrogen in a drybox using standard three-electrode techniques. Experiments were conducted in a vial-based electrochemical setup equipped with a glassy carbon working electrode, a platinum wire counter electrode, and a silver wire pseudoreference electrode. Data were collected using a BASi Epsilon potentiostat. Electrolyte solutions consisted of 0.1 M tetrabutylammonium hexafluorophosphate (TBAPF_6_) in THF. All solutions were prepared using rigorously dried and degassed solvent. Ferrocene was added at the conclusion of each experiment as an internal standard, and all potentials are reported relative to the ferrocene/ferrocenium (Fc/Fc^+^) redox couple. Voltammograms were recorded at room temperature at a scan rate of 100 mV/s unless otherwise noted.

Single crystals suitable for X-ray diffraction were coated with polyisobutylene oil in the drybox, transferred to a nylon loop and then quickly transferred to the goniometer head of a diffractometer equipped with a Bruker PHOTON III detector and Cu X-ray tube (λ = 1.54178 Å). Preliminary data revealed the crystal system. The data collection strategy was optimized for completeness and redundancy using the Bruker APEXII software suite. The space group was identified, and the data were processed and corrected for absorption. The structures were solved using intrinsic phasing (SHELXT) and completed by subsequent Fourier synthesis and refined by full-matrix least-squares procedures in Olex2. Unless otherwise specified, hydrogen atoms were modeled as riding atoms.

### Preparation of Compounds

The following compounds were made according to literature procedures: triphenyl methyl azide,^40^ Ferrocenium tetrakis­[3,5-bis­(trifluoromethyl)-phenyl]­borate,^41^ Pentamethyl ferrocenium tetrakis­(2,3,4,5,6-pentafluorophenyl)­borate,^42^ bis­(bis­(diethylphosphino)­ethane) chromium dichloride.^43^

### Preparation of Cr1

In a nitrogen-filled glovebox, a 150 mL thick-walled vessel was charged with 1.874 g (3.500 mmol, 1.000 equiv) of bis­(bis­(diethylphosphino)­ethane)chromium dichloride, 0.425 g (17.5 mmol, 5.00 equiv) of magnesium powder, and a Teflon coated stirbar. To this mixture was added 40 mL of THF resulting in a yellow-green solution with a metallic suspension. The vessel was sealed and the contents stirred for 16 h whereupon it was opened and refilled with nitrogen and subsequently sealed again and stirred another 24 h. The now red solution with a black and metallic suspension was exposed to low pressure and the volatiles removed. The resulting solid was extracted with 60 mL of toluene and the volatiles were again removed under reduced pressure, resulting in a microcrystalline red solid. The solid was dissolved in a minimal amount of toluene, layered with pentane and placed in a −30 °C freezer. After 48 h red crystals had formed and the solid was collected on a medium porosity fritted filter and washed with 3 × 4 mL of cold pentane. Yield 58%, 1.048 g of **Cr1**. Single crystals suitable for X-ray diffraction were obtained by recrystallization from saturated pentane solutions at −30 °C. Spectroscopic data matched those reported previously.^24^

### Preparation of Cr1-Benz

In a nitrogen-filled glovebox, a 150 mL thick walled glass vessel was charged with 0.245 g (1.99 mmol, 1.00 equiv) of CrCl_2_, 1.119 g (5.646 mmol, 2.84 equiv) of dep-benz, and a Teflon coated stir bar. To this mixture was added 40 mL of THF resulting in a white suspension. The vessel was sealed, removed from the glovebox, and stirred for 2 h in an oil bath at 80 °C. The now red solution was cooled to room temperature which resulted in the deposition of red crystals. The vessel was transferred back into a nitrogen-filled glovebox, where 0.138 g (6.00 mmol, 3.02 equiv) of Na^0^ was added. The suspension was stirred for 72 h and then the volatiles removed under reduced pressure. The resulting red, black, and metallic solid mixture was triturated with 10 mL of toluene. Subsequently, the solid was extracted with 100 mL of toluene and filtered through a pad of celite on a fine porosity fritted filter. The black and metallic solid was discarded, and the volatiles of the red filtrate were removed under reduced vacuum resulting in a red solid. The solid was dissolved in a minimal amount of toluene, layered with pentane and placed in a −30 °C freezer. After 48 h red crystals had formed and the solid was collected on a medium porosity fritted filter and washed with 3 × 4 mL of cold pentane. A second crop of crystals was obtained by removal of the volatiles of the mother liquor under reduced pressure and repeating the crystallization conditions. Failure to wash the product sufficiently results in cocrystallization with sodium chloride. Yield 37%, 0.456 g of **Cr1-benz**. Single crystals suitable for X-ray diffraction analysis were obtained by recrystallization from saturated pentane solutions at −30 °C. Anal. Calcd for C_28_H_48_CrN_4_P_4_: C, 54.54; H, 7.85; N, 9.09. Found: C, 54.61; H, 7.65; N, 9.94. ^1^H NMR (400 MHz, C_6_D_6_): δ 7.64 (d, *J* = 3.8 Hz, 4H), 7.23 (dd, *J* = 5.5, 3.5 Hz, 4H), 2.44 (dq, *J* = 15.2, 7.7 Hz, 8H), 2.11 (dq, *J* = 15.1, 7.7 Hz, 8H), 1.11 (dt, *J* = 12.5, 7.7 Hz, 24H). ^13^C­{^1^H} NMR (101 MHz, C_6_D_6_): δ 146.88, 129.34, 127.44 (overlapping with C_6_D_6_), 24.93, 9.61. ^31^P­{^1^H} NMR (162 MHz, C_6_D_6_): δ 78.41. IR (pentane): ν­(Cr–N≡N) = 1930 cm^–1^.

### Preparation of Cr2

In a nitrogen-filled glovebox, a 20 mL scintillation vial was charged with 0.520 g (0.999 mmol, 1.00 equiv) of **Cr1**, 5 mL of Et_2_O and a Teflon coated stir bar resulting in a red solution. A separate 20 mL scintillation vial was charged with 1.050 g (1.001 mmol, 1.00 equiv) of FcBAr^F^_4_ and 10 mL of Et_2_O, which were mixed until a homogeneous blue solution formed. To the stirring solution of **Cr1** the solution of FcBAr^F^_4_ was added dropwise. The blue color of the Fc^+^ solution did not persist upon addition and was immediately quenched, yielding a yellow–orange solution at the end of the addition. Care should be taken to avoid excess Fc^+^, as addition of 2 equiv or more produces an unidentified, paramagnetic blue chromium complex; if the solution remains green during addition, further Fc^+^ addition should be halted. The volatiles of the solution were removed under reduced pressure. The resulting yellow-orange solid was washed with 3 × 4 mL of pentane. The remaining solid was dissolved in a minimal amount of diethyl ether, filtered through a celite pad on top of a medium porosity fritted filter, and the solution was layered with pentane and placed in a −30 °C freezer. After 24 h orange crystals had formed and the solid was collected on a medium porosity fritted filter and washed 3 × 4 mL with cold pentane. Yield 92%, 1.227 g of **Cr2**. Magnetic susceptibility (Evans Method, THF-*d*_8_, 23 °C): μ_eff_ = 5.6(1) μ_B_. Anal. Calcd for C_52_H_60_BCrF_24_P_4_: C, 47.04; H, 4.56; N, 0.00. Found: C, 47.14; H, 4.27; N, <0.10.

### Preparation of **Cr2′**

In a nitrogen-filled glovebox a 20 mL scintillation vial was charged with 0.052 g (0.10 mmol, 1.0 equiv) of **Cr1**, and 5 mL of Et_2_O, and a Teflon coated stirbar and a red solution was observed. A separate 20 mL scintillation vial was charged with 0.100 g (0.0994 mmol, 1.0 equiv) of Fc*BAr^F^_20_ and 5 mL of THF, which were mixed until a homogeneous blue solution formed. To the stirring solution of **Cr1** the solution of Fc*BAr^F^_20_ was added dropwise. The green color of the Fc^+^ solution did not persist upon addition and was immediately quenched, yielding a yellow–orange solution at the end of the addition. The volatiles of the solution were removed under reduced pressure. The resulting yellow-orange solid was washed with 3 × 4 mL of diethyl ether. The remaining solid was dissolved in minimal THF, filtered through a celite pad on top of a medium porosity fritted filter, and the solution was layered with pentane and placed in a −30 °C freezer. After 24 h orange crystals had formed and the solid was collected on a medium porosity fritted filter and washed with 3 × 4 mL of cold pentane. X-ray quality crystals were obtained from a THF solution of **Cr2′** layered with pentane. Yield 82%, 0.094 g of **Cr2′**. Anal. Calcd for C_44_H_48_BCrF_20_P_4_: C, 46.21; H, 4.23; N, 0.00. Found: C, 46.21; H, 4.17; N, <0.10.

### Preparation of Cr2-Benz

In a nitrogen-filled glovebox, a 20 mL scintillation vial was charged with 0.062 g (0.10 mmol, 1.0 equiv) of **Cr1-benz**, 5 mL of Et_2_O, and a Teflon coated stir bar and a red solution was observed. A separate 20 mL scintillation vial was charged with 0.105 g (0.100 mmol, 1.0 equiv) of FcBAr^F^_4_ and 5 mL of Et_2_O, which were mixed until a homogeneous blue solution formed. To the stirring suspension of **Cr1-benz** the solution of FcBAr^F^_4_ was added dropwise. The blue color of the Fc^+^ solution did not persist upon addition and was immediately quenched, yielding a yellow–orange solution by the end of the addition. Care should be taken to avoid excess Fc^+^, as addition of 2 equiv or more produces an unidentified, paramagnetic blue chromium complex; if the solution remains green during addition, further Fc^+^ addition should be halted. The volatiles of the solution were removed under reduced pressure. The resulting yellow-orange solid was washed with 3 × 4 mL of diethyl ether. The remaining solid was dissolved in a minimal amount of THF, filtered through a celite pad on top of a medium porosity fritted filter, and the solution was layered with pentane and placed in a −30 °C freezer. After 24 h orange crystals were obtained and the solid was collected on a medium porosity fritted filter and washed 3 × 4 mL with cold pentane. X-ray quality crystals were obtained from solutions of **Cr2-benz** in THF layered with pentane. Yield 87%, 0.124 g of **Cr2-benz**. Magnetic susceptibility (Evans Method, THF-*d*_8_, 23 °C): μ_eff_ = 5.6(3) μ_B_. Anal. Calcd for C_60_H_60_CrP_4_BF_24_: C, 50.62; H, 4.25; N, 0.00. Found: C, 50.05; H, 4.15; N, <0.10.

### Preparation of Cr3

In a nitrogen-filled glovebox a 20 mL scintillation vial was charged with 0.664 g (0.500 mmol, 1.00 equiv) of **Cr2**, 10 mL of Et_2_O and a Teflon coated stir bar and a yellow solution formed. To this solution, 0.143 g (0.501 mmol, 1.00 equiv) of solid Ph_3_CN_3_ were added resulting in gas evolution and a color change to green. The volatiles of the solution were removed under reduced pressure. The resulting yellow-green solid was washed with 3 × 3 mL of 1:2 toluene/pentane. The remaining solid was dissolved in minimal Et_2_O, filtered through a celite pad on top of a medium porosity fritted filter, and the solution was layered with pentane and placed in a −30 °C freezer. After 24 h green crystals were obtained and the green solid was collected on a medium porosity fritted filter and washed with 3 × 4 mL cold pentane. X-ray quality crystals were obtained from solutions of **Cr3** in diethyl ether layered with pentane. Yield 79%, 0.532 g of **Cr3**. **Cr3-** ^15^ **N** can be made using an analogous method using 50% α, 50% γ ^15^N labeled Ph_3_CN_3_. Anal. Calcd for C_52_H_60_BCrF_24_NP_4_: C, 46.55; H, 4.51; N, 1.04. Found: C, 45.94; H, 4.20; N, 0.89. ^1^H NMR (400 MHz, THF-*d*_8_): δ 7.95–7.71 (m, 8H), 7.61 (s, 4H), 2.43 (dh, *J* = 11.7, 7.4 Hz, 4H), 2.22–1.90 (m, 16H), 1.64 (dq, *J* = 15.1, 7.6 Hz, 4H), 1.32 (dt, *J* = 15.3, 7.5 Hz, 12H), 1.04–0.86 (m, 12H). ^13^C­{^1^H} NMR (101 MHz, THF-*d*_8_): δ 161.83 (q, *J* = 50 Hz), 134.62, 129.05 (q, *J* = 32 Hz), 124.53 (q, *J* = 272.0 Hz), 117.19, 22.23, 21.90, 21.72, 18.42, 7.63, 7.31. ^31^P­{^1^H} NMR (162 MHz, THF-*d*_8_): δ 76.18. ^19^F­{^1^H} NMR (367 MHz, THF-*d*_8_): δ −63.41. ^15^N (41 MHz, THF-*d*_8_): δ 1026.08.

### Preparation of Cr3-Benz

In a nitrogen-filled glovebox, a 20 mL scintillation vial was charged with 0.062 g (0.10 mmol, 1.0 equiv) of **Cr1-benz**, 5 mL of Et_2_O and a Teflon coated stir bar forming a red solution. A separate 20 mL scintillation vial was charged with 0.105 g (0.100 mmol, 1.0 equiv) of FcBAr^F^_4_ and 5 mL of Et_2_O, which were mixed until a homogeneous blue solution formed. To the stirring suspension of **Cr1-benz** the solution of FcBAr^F^_4_ was added dropwise. The blue color of the Fc^+^ solution did not persist upon addition and was immediately quenched, yielding a yellow–orange solution at the end of the addition. Care should be taken to avoid excess Fc^+^, as addition of 2 equiv or more produces an unidentified, paramagnetic blue chromium complex; if the solution remains green during addition, further Fc^+^ addition should be halted. To this solution, solid Ph_3_CN_3_ was added resulting in gas evolution and a color change to green was observed. The volatiles of the solution were removed under reduced pressure. The resulting yellow-green solid was washed with 3 × 3 mL of 1:2 toluene/pentane. The remaining solid was dissolved in minimal THF, filtered through a celite pad on top of a medium porosity fritted filter, and the solution was layered with pentane and placed in a −30 °C freezer. After 48 h green crystals had formed and were collected on a medium porosity fritted filter and washed with 3 × 4 mL of cold pentane. X-ray quality crystals were obtained from solutions of **Cr3-benz** in THF layered with pentane. Yield 91%, 0.131 g of **Cr3-benz**. Anal. Calcd for C_60_H_60_BCrF_24_NP_4_: C, 50.12; H, 4.21; N, 0.97. Found: C, 49.92; H, 3.79; N, 0.83. ^1^H NMR (400 MHz, THF): δ 8.18 (t, *J* = 3.6 Hz, 4H), 7.83 (dd, *J* = 5.6, 3.0 Hz, 12H), 7.61 (s, 4H), 2.98 (dq, *J* = 14.7, 7.3 Hz, 4H), 2.52 (dq, *J* = 14.7, 7.3 Hz, 4H), 2.34 (dq, *J* = 15.1, 7.6 Hz, 4H), 2.06 (dq, *J* = 15.1, 7.6 Hz, 4H), 1.04 (p, *J* = 7.5 Hz, 4H), 0.67 (p, *J* = 7.5 Hz, 4H). ^13^C­{^1^H} NMR (101 MHz, THF-*d*_8_): δ 161.83 (q, *J* = 50 Hz), 142.64, 134.62, 131.25, 130.12, 129.05 (q, *J* = 32 Hz), 124.53 (q, *J* = 272.0 Hz), 117.19, 22.98, 22.75, 8.99, 6.89. ^31^P­{^1^H} NMR (162 MHz, THF-*d*_8_): δ 78.69. ^19^F­{^1^H} NMR (367 MHz, THF-*d*_8_): δ −63.41.

### Formation of Cr4

In a nitrogen-filled glovebox, a J. Young NMR tube was charged with 0.013 g (0.0097 mmol, 1.0 equiv) of **Cr3** and 0.5 mL of THF-*d*_8_ resulting in a green solution. To this solution, 0.2 mL of a stock solution of 2,6-dimethylphenyl isocyanide, consisting of 0.006 g of 2,6-dimethylphenyl isocyanide (0.05 mmol) and 1 mL of THF-*d*_8_, were added and a color change to red was. The ^31^P­{^1^H} NMR spectrum was recorded and the J. Young NMR tube was returned to the glovebox. The volatiles were removed under reduced pressure. The red residue was dissolved in 2 mL of diethyl ether, layered with pentane, and placed in a −30 °C freezer. This first results in an oil which over 7 days results in red crystals suitable for X-ray diffraction. ^31^P­{^1^H} NMR (162 MHz, THF-*d*_8_): δ 72.13 (dd, *J*_P2‑P4_ = 6.2 Hz, *J*_P3‑P4_ = 20.1 Hz, 1P): δ 60.89 (dd, *J*_P2‑P3_ = 34.7 Hz, *J*_P3‑P4_ = 19.8 Hz, 1P): δ 55.98 (ddd, *J*_P1‑P2_ = 28.2 Hz, *J*_P2‑P3_ = 34.7 Hz, *J*_P2‑P4_ = 6.4 Hz, 1P): δ 5.52 (d, *J*_P1‑P2_ = 28.2 Hz, 1P).

### Addition of AdNC

In a nitrogen-filled glovebox, a J. Young NMR tube was charged with 0.013 g (0.0097 mmol, 1.0 equiv) of **Cr3** and 0.5 mL of THF-*d*_8_, resulting in a green solution. To this solution 0.2 mL of a stock solution of 1-adamantyl isocyanide, consisting of 0.008 g of 1-adamantyl isocyanide (0.05 mmol) and 1 mL of THF-*d*_8_, were added and a color change to red was observed. A ^31^P­{^1^H} NMR spectrum was then recorded. ^31^P­{^1^H} NMR (162 MHz, THF-*d*_8_): δ 72.96 (dd, *J*_PP_ = 5.0 Hz, *J*_PP_ = 16.8 Hz, 1P): δ 72.61 (s, 4P): δ 62.98 (dd, *J*_PP_ = 31.7 Hz, *J*_PP_ = 16.8 Hz, 1P): δ 57.05 (td, *J*_PP_ = 31.0 Hz, *J*_PP_ = 5.0 Hz, 1P): δ −7.62 (d, *J*_PP_ = 30.2 Hz, 1P).

## Supplementary Material



## Notes

Preparation of Compounds; NMR Spectra; EPR Spectra; X-ray Crystallographic Data; Electrochemical Data; IR Spectra (PDF)
