# Supplementary material for: Synthesis of Chromium(IV) Nitrides Through High-Spin Tetrahedral Chromium(I) Intermediates
Source: Inorg Chem. 2026 Feb 19;65(9):5156–65. doi: 10.1021/acs.inorgchem.5c05944 (PMC12977042; doi:10.1021/acs.inorgchem.5c05944)
Supplement: Supplementary file 1 [file ic5c05944_si_001.pdf]

Supporting Information

Synthesis of Chromium(IV) Nitrides Through High-Spin Tetrahedral Chromium(I) Intermediates

*Grace B. Panetti, Matthew V. Pecoraro, Runzi Li, Junho Kim, Gabriele Hierlmeier, Paul J. Chirik**

Department of Chemistry, Frick Laboratory
Princeton University, Princeton, NJ 08544, USA

pchirik@princeton.edu

Table of Contents

I. General Considerations S2

II. Preparation of Compounds S5

III. NMR Spectra S12

IV. EPR Spectra S24

V. X-Ray Crystallographic Data S26

VI. Electrochemical Data S38

VII. IR Spectra S39

VIII. References S40

I. General Considerations

All air- and moisture-sensitive manipulations were carried out using vacuum line, Schlenk and cannula techniques or in an MBraun inert atmosphere nitrogen dry box unless otherwise noted. All glassware was stored in a pre-heated oven (≥ 150° C) prior to use. Pentane, benzene, toluene, diethyl ether, and tetrahydrofuran used for air- and moisture-sensitive manipulations were dried and deoxygenated using literature procedures.[1] Benzene-d_6 used for NMR spectroscopy was distilled from sodium metal and stored over 4 Å molecular sieves. THF-d_8 used for NMR spectroscopy was dried using sodium-benzophenone ketyl[2] and directly vacuum transferred to the reaction mixtures prior to use. celite, alumina, and silica were dried at 180 ºC under vacuum for 3 days prior to use in the glovebox. Solid reagents were dried under vacuum overnight and stored under dinitrogen prior to use. All compounds were purchased from Sigma Aldrich, Alfa Aesar, Tokyo Chemical Industry, and Acros Organics and used as received unless otherwise stated.

^{1}H NMR spectra were recorded on either Bruker AVANCE 300, 400 or 500 spectrophotometers operating at 300.13 MHz, 399.8 MHz and 500.46 MHz, respectively. ^{13}C NMR spectra were recorded on either Bruker Avance 300, 400 or 500 spectrometers operating at 75.48 MHz, 100.54 MHz and 125.85 MHz, respectively. All ^{1}H and ^{13}C NMR chemical shifts are reported in ppm relative to $SiMe_4$ using the ^{1}H and ^{13}C chemical shifts of the solvent[3] as a standard. ^{1}H NMR data for diamagnetic compounds are reported as follows: chemical shift, multiplicity (s = singlet, d = doublet, t = triplet, q = quartet, p = pentet, br = broad, m = multiplet, app = apparent, obsc = obscured), coupling constants (Hz), integration, assignment. ^{13}C NMR data for diamagnetic compounds are reported as follows: chemical shift, number of protons attached to carbon (e.g. CH_2), assignment. ^{2}H NMR spectra were recorded on Bruker Avance 400 or 500 spectrometers operating at 61.42 MHz and 76.88 MHz, respectively and referenced to TMS-d_{12} as an external standard. ^{19}F NMR spectra were recorded on Bruker Avance 400 or 500 spectrometers operating at 376.19 MHz and 470.96 MHZ, respectively, and referenced to

$CFCl_3$ as an external standard. ^{31}P NMR spectra were recorded on either Bruker Avance 400 or 500 spectrometers operating at 161.84 MHz and 202.00 MHz, respectively, and were referenced to 85% H_3PO_4 as an external standard. ^{15}N NMR spectra were recorded on a Bruker Avance 400 spectrometer operating at 40.51 MHz and referenced to $^{15}NH_3$ as an external standard.

Continuous wave EPR spectra were recorded at room temperature or 20 K on an X-band Bruker EMXPlus spectrometer equipped with an EMX standard resonator and a Bruker PremiumX microwave bridge. The spectra were simulated using EasySpin for MATLAB.[4] Elemental analyses were performed at Robertson Microlit Laboratories, Inc., in Ledgewood, NJ. Infrared spectroscopy was conducted on a Thermo-Nicolet iS10 FT-IR spectrometer calibrated with a polystyrene standard.

Cyclic voltammetry measurements were performed under an inert atmosphere of nitrogen in a drybox using standard three-electrode techniques. Experiments were conducted in a vial-based electrochemical setup equipped with a glassy carbon working electrode, a platinum wire counter electrode, and a silver wire pseudo-reference electrode. Data were collected using a BASi Epsilon potentiostat. Electrolyte solutions consisted of 0.1 M tetrabutylammonium hexafluorophosphate ($TBAPF_6$) in THF. All solutions were prepared using rigorously dried and degassed solvent. Ferrocene was added at the conclusion of each experiment as an internal standard, and all potentials are reported relative to the ferrocene/ferrocenium (Fc/Fc^+) redox couple. Voltammograms were recorded at room temperature at a scan rate of 100 mV/s unless otherwise noted.

Single crystals suitable for X-ray diffraction were coated with polyisobutylene oil in the drybox, transferred to a nylon loop and then quickly transferred to the goniometer head of a diffractometer equipped with a Bruker PHOTON III detector and Cu X-Ray tube (λ = 1.54178 Å). Preliminary data revealed the crystal system. The data collection strategy was optimized for completeness and redundancy using either the Bruker APEXII software suite. The space group

was identified, and the data were processed and corrected for absorption. The structures were solved using intrinsic phasing (SHELXT) and completed by subsequent Fourier synthesis and refined by full-matrix least-squares procedures in Olex2. Unless otherwise specified, hydrogen atoms were modelled as riding atoms.

II. Preparation of compounds

The following compounds were made according to literature procedures: triphenyl methyl azide,[5] Ferrocenium tetrakis[3,5-bis(trifluoromethyl)phenyl]borate,[6] Pentamethyl ferrocenium tetrakis(2,3,4,5,6-pentafluorophenyl)borate,[7] bis(bis(diethylphosphino)ethane) chromium dichloride [8].

Preparation of Cr1. In a nitrogen-filled glovebox, a 150 mL thick-walled vessel was charged with 1.874 g (3.500 mmol, 1.000 equiv) of bis(bis(diethylphosphino)ethane)chromium dichloride, 0.425 g (17.5 mmol, 5.00 equiv) of magnesium powder, and a Teflon coated stirbar. To this mixture was added 40 mL of THF resulting in a yellow-green solution with a metallic suspension. The vessel was sealed and the contents stirred for 16 hours whereupon it was opened and refilled with nitrogen and subsequently sealed again and stirred another 24 hours. The now red solution with a black and metallic suspension was exposed to low pressure and the volatiles removed. The resulting solid was extracted with 60 mL of toluene and the volatiles were again removed under reduced pressure, resulting in a microcrystalline red solid. The solid was dissolved in a minimal amount of toluene, layered with pentane and placed in a –30 °C freezer. After 48 h red crystals had formed and the solid was collected on a medium porosity fritted filter and washed with 3 x 4 mL of cold pentane. Yield 58%, 1.048 g of **Cr1**. Single crystals suitable for X-ray diffraction were obtained by recrystallization from saturated pentane solutions at –30 °C. Spectroscopic data matched those reported previously.[9]

Preparation of Cr1-benz. In a nitrogen-filled glovebox, a 150 mL thick walled glass vessel was charged with 0.245 g (1.99 mmol, 1.00 equiv) of $CrCl_2$, 1.119 g (5.646 mmol, 2.84 equiv) of dep-benz, and a Teflon coated stir bar. To this mixture was added 40 mL of THF resulting in a white suspension. The vessel was sealed, removed from the glovebox, and stirred for 2 h in an oil

bath at 80 °C. The now red solution was cooled to room temperature which resulted in the deposition of red crystals. The vessel was transferred back into a nitrogen-filled glovebox, where 0.138 g (6.00 mmol, 3.02 equiv) of Na^0 was added. The suspension was stirred for 72 hours and then the volatiles removed under reduced pressure. The resulting red, black, and metallic solid mixture was triturated with 10 mL of toluene. Subsequently, the solid was extracted with 100 mL of toluene and filtered through a pad of celite on a fine porosity fritted filter. The black and metallic solid was discarded, and the volatiles of the red filtrate were removed under reduced vacuum resulting in a red solid. The solid was dissolved in a minimal amount of toluene, layered with pentane and placed in a –30 °C freezer. After 48 h red crystals had formed and the solid was collected on a medium porosity fritted filter and washed with 3 x 4 mL of cold pentane. A second crop of crystals was obtained by removal of the volatiles of the mother liquor under reduced pressure and repeating the crystallization conditions. Failure to wash the product sufficiently results in co-crystallization with sodium chloride. Yield 37%, 0.456 g of **Cr1-benz**. Single crystals suitable for X-ray diffraction analysis were obtained by recrystallization from saturated pentane solutions at –30 °C. Anal Calcd. for $C_{28}H_{48}CrN_4P_4$: C, 54.54; H, 7.85; N, 9.09. Found: C, 54.61; H, 7.65; N, 9.94. ^1H NMR (400 MHz, C_6D_6) δ 7.64 (d, J = 3.8 Hz, 4H), 7.23 (dd, J = 5.5, 3.5 Hz, 4H), 2.44 (dq, J = 15.2, 7.7 Hz, 8H), 2.11 (dq, J = 15.1, 7.7 Hz, 8H), 1.11 (dt, J = 12.5, 7.7 Hz, 24H). $^{13}C\{^1H\}$ NMR (101 MHz, C_6D_6) δ 146.88, 129.34, 127.44 (overlapping with C_6D_6), 24.93, 9.61. $^{31}P\{^1H\}$ NMR (162 MHz, C_6D_6) δ 78.41. IR (pentane): v(Cr-N≡N) = 1930 cm^{-1}.

Preparation of Cr2. In a nitrogen-filled glovebox, a 20 mL scintillation vial was charged with 0.520 g (0.999 mmol, 1.00 equiv) of **Cr1**, 5 mL of Et_2O and a Teflon coated stir bar resulting in a red solution. A separate 20 mL scintillation vial was charged with 1.050 g (1.001 mmol, 1.00 equiv) of $FcBAr^F_4$ and 10 mL of Et_2O, which were mixed until a homogenous blue solution

formed. To the stirring solution of **Cr1** the solution of $FcBAr^F_4$ was added dropwise. The blue color of the Fc^+ solution did not persist upon addition and was immediately quenched, yielding a yellow–orange solution at the end of the addition. Care should be taken to avoid excess Fc^+, as addition of 2 equiv or more produces an unidentified, paramagnetic blue chromium complex; if the solution remains green during addition, further Fc^+ addition should be halted. The volatiles of the solution were removed under reduced pressure. The resulting yellow-orange solid was washed with 3 x 4 mL of pentane. The remaining solid was dissolved in a minimal amount of diethyl ether, filtered through a celite pad on top of a medium porosity fritted filter, and the solution was layered with pentane and placed in a –30°C freezer. After 24 h orange crystals had formed and the solid was collected on a medium porosity fritted filter and washed 3 x 4 mL with cold pentane. Yield 92%, 1.227 g of **Cr2**. Magnetic susceptibility (Evans Method, THF-d_8, 23 °C): μ_{eff} = 5.6(1) μ_B. Anal Calcd. for $C_{52}H_{60}BCrF_{24}P_4$: C, 47.04; H, 4.56; N, 0.00. Found: C, 47.14; H, 4.27; N, <0.10.

Preparation of Cr2´. In a nitrogen-filled glovebox a 20 mL scintillation vial was charged with 0.052 g (0.10 mmol, 1.0 equiv) of **Cr1**, and 5 mL of Et_2O, and a Teflon coated stirbar and a red solution was observed. A separate 20 mL scintillation vial was charged with 0.100 g (0.0994 mmol, 1.0 equiv) of $Fc^*BAr^F_{20}$ and 5 mL of THF, which were mixed until a homogenous blue solution formed. To the stirring solution of **Cr1** the solution of $Fc^*BAr^F_{20}$ was added dropwise. The green color of the Fc^+ solution did not persist upon addition and was immediately quenched, yielding a yellow–orange solution at the end of the addition. The volatiles of the solution were removed under reduced pressure. The resulting yellow-orange solid was washed with 3 x 4 mL of diethyl ether. The remaining solid was dissolved in minimal THF, filtered through a celite pad on top of a medium porosity fritted filter, and the solution was layered with pentane and placed in a -30 °C freezer. After 24 h orange crystals had formed and the solid was

collected on a medium porosity fritted filter and washed with 3 x 4 mL of cold pentane. X-ray quality crystals were obtained from a THF solution of **Cr2´** layered with pentane. Yield 82%, 0.094 g of **Cr2´**. Anal Calcd. for $C_{44}H_{48}BCrF_{20}P_4$: C, 46.21; H, 4.23; N, 0.00. Found: C, 46.21; H, 4.17; N, <0.10.

Preparation of Cr2-benz. In a nitrogen-filled glovebox, a 20 mL scintillation vial was charged with 0.062 g (0.10 mmol, 1.0 equiv) of **Cr1-benz**, 5 mL of Et_2O, and a Teflon coated stir bar and a red solution was observed. A separate 20 mL scintillation vial was charged with 0.105 g (0.100 mmol, 1.0 equiv) of $FcBAr^F_4$ and 5 mL of Et_2O, which were mixed until a homogenous blue solution formed. To the stirring suspension of **Cr1-benz** the solution of $FcBAr^F_4$ was added dropwise. The blue color of the Fc^+ solution did not persist upon addition and was immediately quenched, yielding a yellow–orange solution by the end of the addition. Care should be taken to avoid excess Fc^+, as addition of 2 equiv or more produces an unidentified, paramagnetic blue chromium complex; if the solution remains green during addition, further Fc^+ addition should be halted. The volatiles of the solution were removed under reduced pressure. The resulting yellow-orange solid was washed with 3 x 4 mL of diethyl ether. The remaining solid was dissolved in a minimal amount of THF, filtered through a celite pad on top of a medium porosity fritted filter, and the solution was layered with pentane and placed in a –30 °C freezer. After 24 h orange crystals were obtained and the solid was collected on a medium porosity fritted filter and washed 3 x 4 mL with cold pentane. X-ray quality crystals were obtained from solutions of **Cr2-benz** in THF layered with pentane. Yield 87%, 0.124 g of **Cr2-benz**. Magnetic susceptibility (Evans Method, THF-*d*$_8$, 23 °C): μ_{eff} = 5.6(3) μ_B. Anal Calcd. for $C_{60}H_{60}CrP_4BF_{24}$: C, 50.62; H, 4.25; N, 0.00. Found: C, 50.05; H, 4.15; N, <0.10.

Preparation of Cr3. In a nitrogen-filled glovebox a 20 mL scintillation vial was charged with 0.664 g (0.500 mmol, 1.00 equiv) of **Cr2**, 10 mL of Et_2O and a Teflon coated stir bar and a yellow solution formed. To this solution, 0.143 g (0.501 mmol, 1.00 equiv) of solid Ph_3CN_3 were added resulting in gas evolution and a color change to green. The volatiles of the solution were removed under reduced pressure. The resulting yellow-green solid was washed with 3 x 3 mL of 1:2 toluene:pentane. The remaining solid was dissolved in minimal Et_2O, filtered through a celite pad on top of a medium porosity fritted filter, and the solution was layered with pentane and placed in a –30 °C freezer. After 24 h green crystals were obtained and the green solid was collected on a medium porosity fritted filter and washed with 3 x 4 mL cold pentane. X-ray quality crystals were obtained from solutions of **Cr3** in diethyl ether layered with pentane. Yield 79%, 0.532 g of **Cr3**. **Cr3-^{15}N** can be made using an analogous method using 50% α, 50% γ ^{15}N labeled Ph_3CN_3. Anal Calcd. for $C_{52}H_{60}BCrF_{24}NP_4$: C, 46.55; H, 4.51; N, 1.04. Found: C, 45.94; H, 4.20; N, 0.89. ^{1}H NMR (400 MHz, THF-d_8) δ 7.95 – 7.71 (m, 8H), 7.61 (s, 4H), 2.43 (dh, J = 11.7, 7.4 Hz, 4H), 2.22 – 1.90 (m, 16H), 1.64 (dq, J = 15.1, 7.6 Hz, 4H), 1.32 (dt, J = 15.3, 7.5 Hz, 12H), 1.04 – 0.86 (m, 12H). ^{13}C{^{1}H} NMR (101 MHz, THF-d_8) δ 161.83 (q, J = 50 Hz), 134.62, 129.05 (q, J = 32 Hz), 124.53 (q, J = 272.0 Hz), 117.19, 22.23, 21.90, 21.72, 18.42, 7.63, 7.31. ^{31}P{^{1}H} NMR (162 MHz, THF-d_8) δ 76.18. ^{19}F{^{1}H} NMR (367 MHz, THF-d_8) δ -63.41. ^{15}N (41 MHz, THF-d_8) δ 1026.08.

Preparation of Cr3-benz. In a nitrogen-filled glovebox, a 20 mL scintillation vial was charged with 0.062 g (0.10 mmol, 1.0 equiv) of **Cr1-benz**, 5 mL of Et_2O and a Teflon coated stir bar forming a red solution. A separate 20 mL scintillation vial was charged with 0.105 g (0.100 mmol, 1.0 equiv) of $FcBAr^F_4$ and 5 mL of Et_2O, which were mixed until a homogenous blue solution formed. To the stirring suspension of **Cr1-benz** the solution of $FcBAr^F_4$ was added dropwise. The blue color of the Fc^+ solution did not persist upon addition and was immediately

quenched, yielding a yellow–orange solution at the end of the addition. Care should be taken to avoid excess Fc^+, as addition of 2 equiv or more produces an unidentified, paramagnetic blue chromium complex; if the solution remains green during addition, further Fc^+ addition should be halted. To this solution, solid Ph_3CN_3 was added resulting in gas evolution and a color change to green was observed. The volatiles of the solution were removed under reduced pressure. The resulting yellow-green solid was washed with 3 x 3 mL of 1:2 toluene:pentane. The remaining solid was dissolved in minimal THF, filtered through a celite pad on top of a medium porosity fritted filter, and the solution was layered with pentane and placed in a –30°C freezer. After 48 h green crystals had formed and were collected on a medium porosity fritted filter and washed with 3 x 4 mL of cold pentane. X-ray quality crystals were obtained from solutions of **Cr3-benz** in THF layered with pentane. Yield 91%, 0.131 g of **Cr3-benz**. Anal Calcd. for $C_{60}H_{60}BCrF_{24}NP_4$: C, 50.12; H, 4.21; N, 0.97. Found: C, 49.92; H, 3.79; N, 0.83. ^{1}H NMR (400 MHz, THF) δ 8.18 (t, *J* = 3.6 Hz, 4H), 7.83 (dd, *J* = 5.6, 3.0 Hz, 12H), 7.61 (s, 4H), 2.98 (dq, *J* = 14.7, 7.3 Hz, 4H), 2.52 (dq, *J* = 14.7, 7.3 Hz, 4H), 2.34 (dq, *J* = 15.1, 7.6 Hz, 4H), 2.06 (dq, *J* = 15.1, 7.6 Hz, 4H), 1.04 (p, *J* = 7.5 Hz, 4H), 0.67 (p, *J* = 7.5 Hz, 4H). ^{13}C{^{1}H} NMR (101 MHz, THF-d_8) δ 161.83 (q, *J* = 50 Hz), 142.64, 134.62, 131.25, 130.12, 129.05 (q, *J* = 32 Hz), 124.53 (q, *J* = 272.0 Hz), 117.19, 22.98, 22.75, 8.99, 6.89. ^{31}P{^{1}H} NMR (162 MHz, THF-d_8) δ 78.69. ^{19}F{^{1}H} NMR (367 MHz, THF-d_8) δ -63.41.

Formation of Cr4. In a nitrogen-filled glovebox, a J. Young NMR tube was charged with 0.013 g (0.0097 mmol, 1.0 equiv) of **Cr3** and 0.5 mL of THF-d_8 resulting in a green solution. To this solution, 0.2 mL of a stock solution of 2,6-dimethylphenyl isocyanide, consisting of 0.006 g of 2,6-dimethylphenyl isocyanide (0.05 mmol) and 1 mL of THF-d_8, were added and a color change to red was. The ^{31}P{^{1}H} NMR spectrum was recorded and the J. Young NMR tube was returned to the glovebox. The volatiles were removed under reduced pressure. The red residue

was dissolved in 2 mL of diethyl ether, layered with pentane, and placed in a –30 °C freezer. This first results in an oil which over 7 days results in red crystals suitable for X-ray diffraction. $^{31}P\{^{1}H\}$ NMR (162 MHz, THF-d_8) δ 72.13 (dd, $J_{P2\text{-}P4}$ = 6.2 Hz, $J_{P3\text{-}P4}$ = 20.1 Hz, 1P), δ 60.89 (dd, $J_{P2\text{-}P3}$ = 34.7 Hz, $J_{P3\text{-}P4}$ = 19.8 Hz, 1P), δ 55.98 (ddd, $J_{P1\text{-}P2}$ = 28.2 Hz, $J_{P2\text{-}P3}$ = 34.7 Hz, $J_{P2\text{-}P4}$ = 6.4 Hz, 1P), δ 5.52 (d, $J_{P1\text{-}P2}$ = 28.2 Hz, 1P).

Addition of AdNC. In a nitrogen-filled glovebox, a J. Young NMR tube was charged with 0.013 g (0.0097 mmol, 1.0 equiv) of **Cr3** and 0.5 mL of THF-d_8, resulting in a green solution. To this solution 0.2 mL of a stock solution of 1-adamantyl isocyanide, consisting of 0.008 g of 1-adamantyl isocyanide (0.05 mmol) and 1 mL of THF-d_8, were added and a color change to red was observed. A $^{31}P\{^{1}H\}$ NMR spectrum was then recorded. $^{31}P\{^{1}H\}$ NMR (162 MHz, THF-d_8) δ 72.96 (dd, J_{PP} = 5.0 Hz, J_{PP} = 16.8 Hz, 1P), δ 72.61 (s, 4P), δ 62.98 (dd, J_{PP} = 31.7 Hz, J_{PP} = 16.8 Hz, 1P), δ 57.05 (td, J_{PP} = 31.0 Hz, J_{PP} = 5.0 Hz, 1P), δ -7.62 (d, J_{PP} = 30.2 Hz, 1P).

III. NMR Spectra

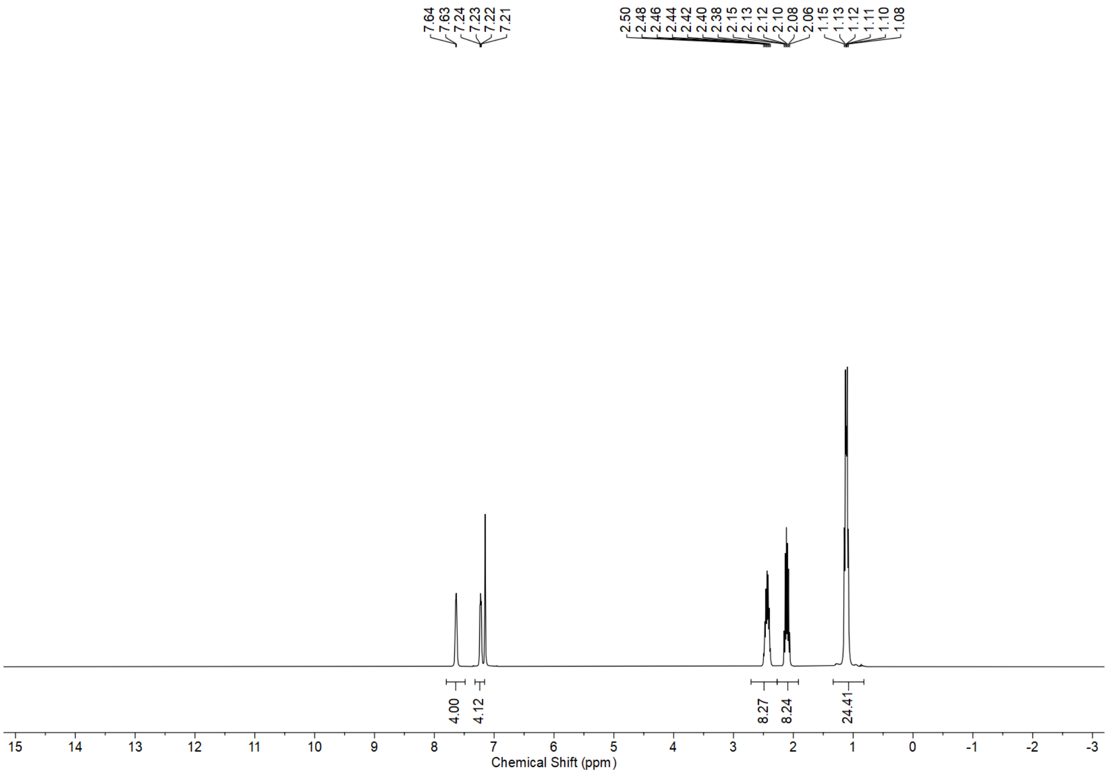

Figure S1. ^{1}H NMR spectrum of **Cr1-benz** in C_6D_6.

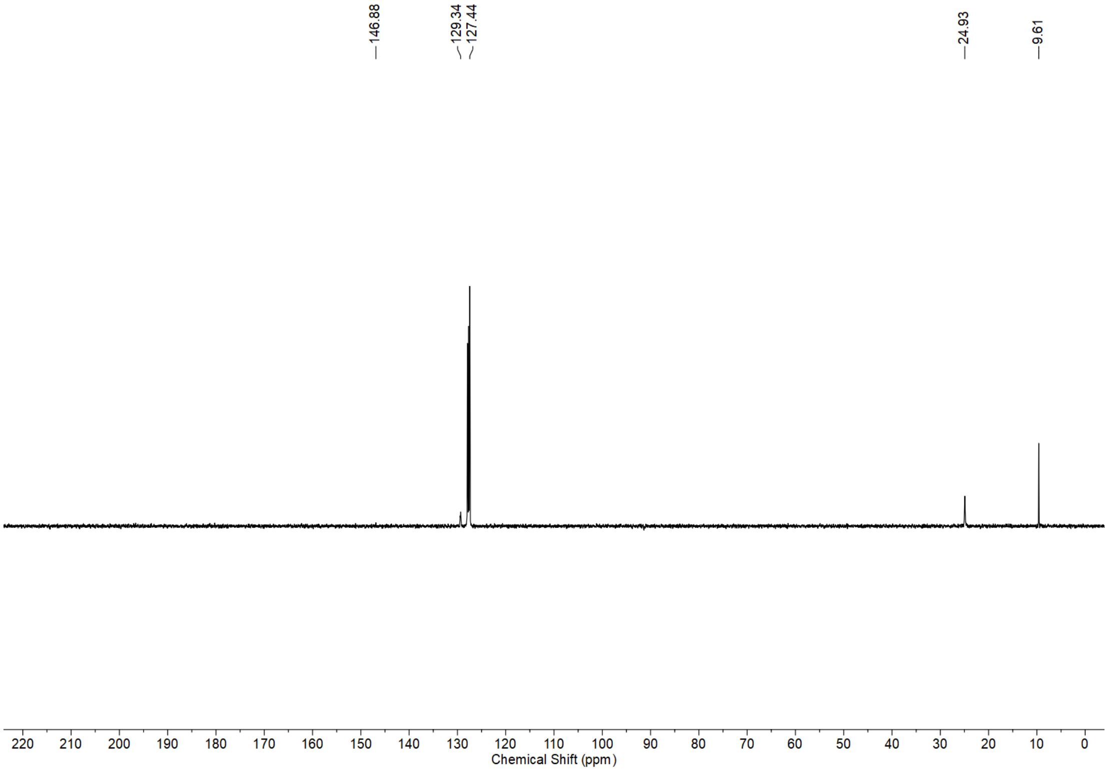

Figure S2. $^{13}C\{^1H\}$ NMR spectrum of **Cr1-benz** in C_6D_6.

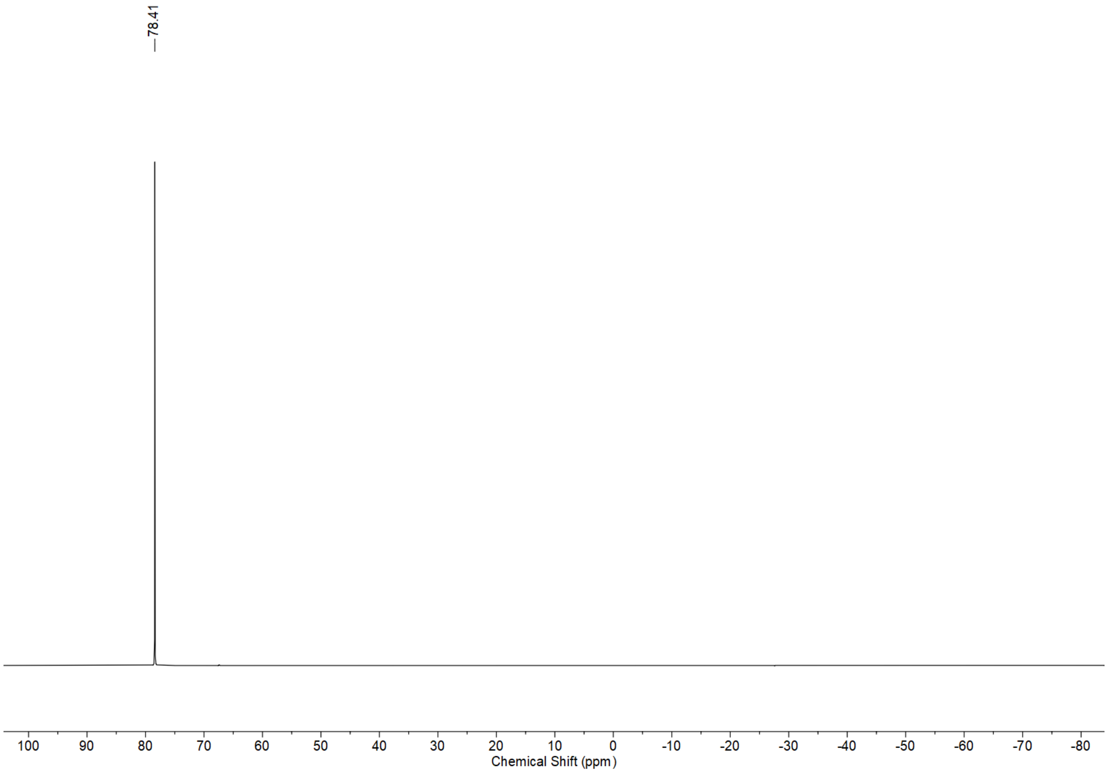

Figure S3. $^{31}P\{^{1}H\}$ NMR spectrum of **Cr1-benz** in C_6D_6.

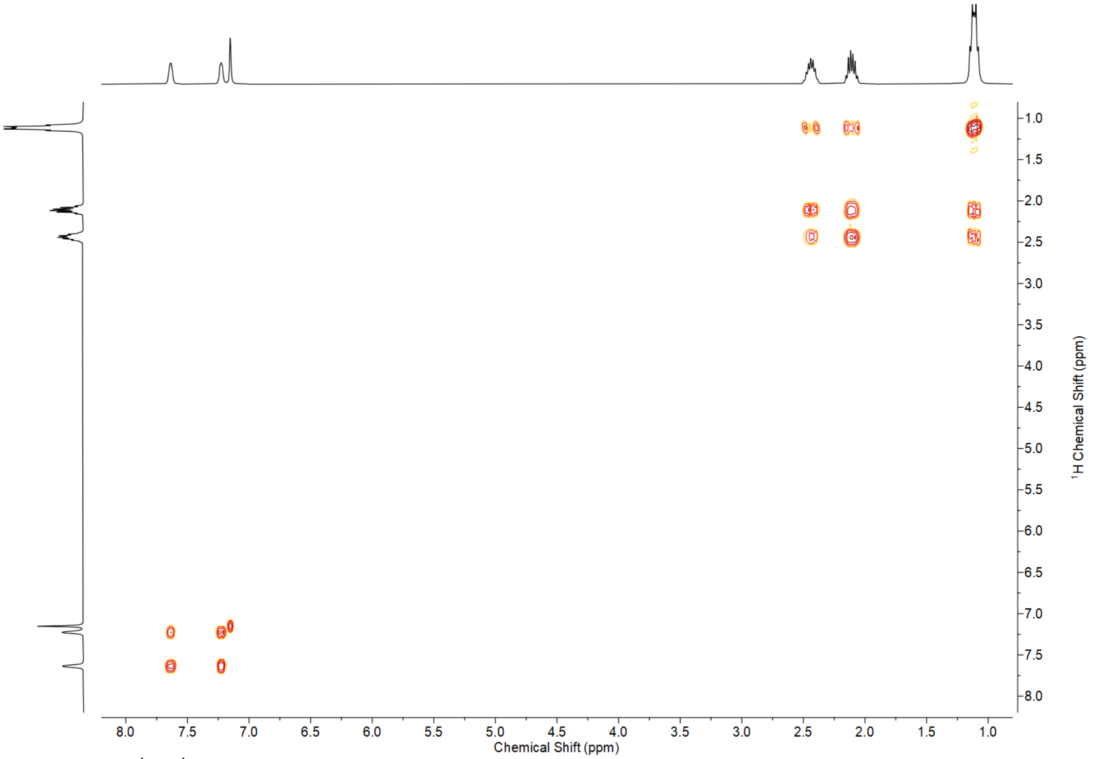

Figure S4. ^{1}H-^{1}H COSY NMR spectrum of **Cr1-benz** in C_6D_6.

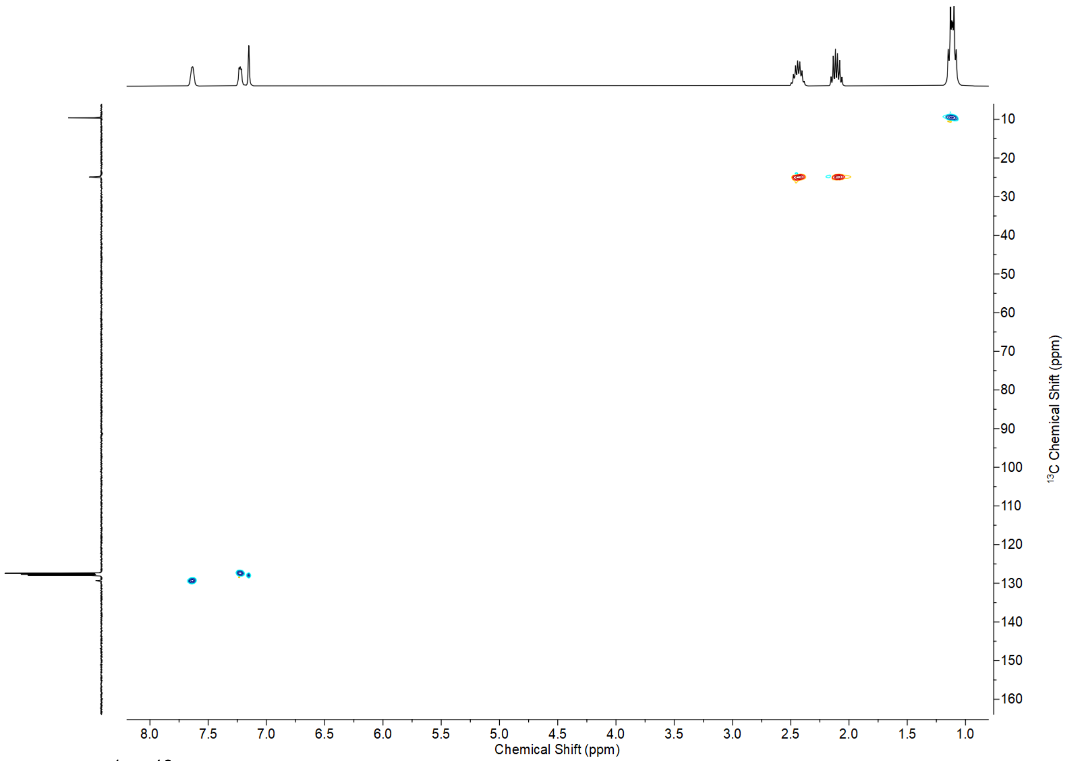

Figure S5. ^{1}H-^{13}C HSQC NMR spectrum of **Cr1-benz** in C_6D_6.

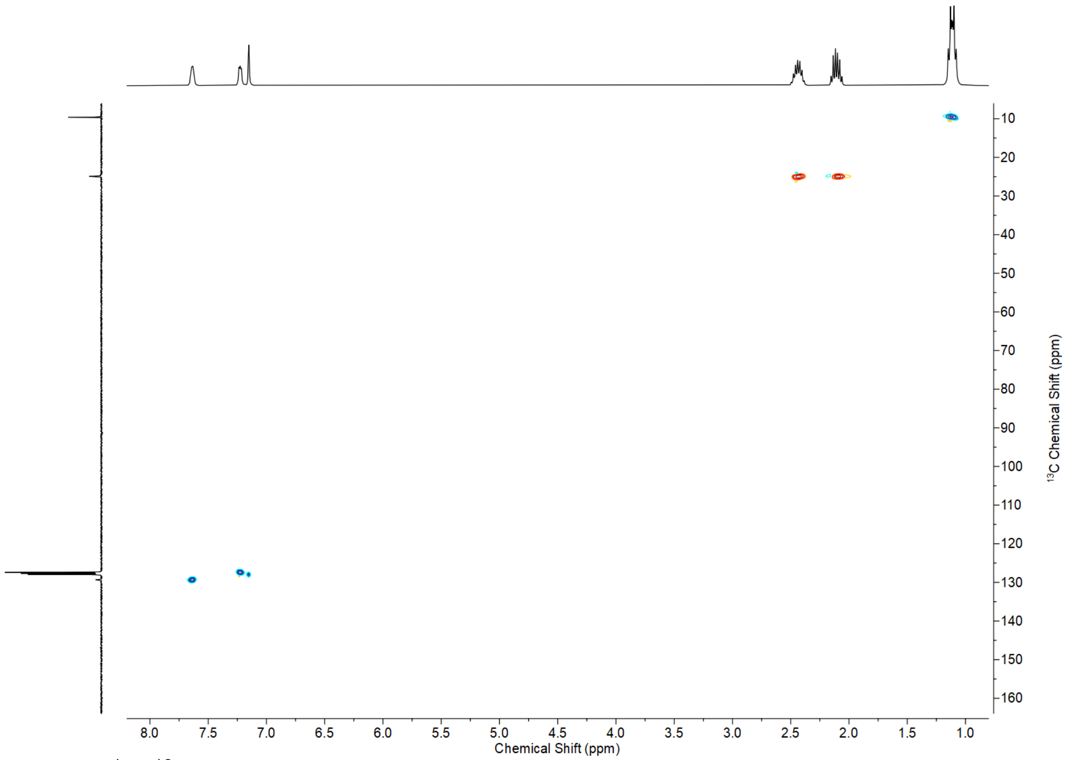

Figure S6. ^{1}H-^{13}C HMBC NMR spectrum of **Cr1-benz** in C_6D_6.

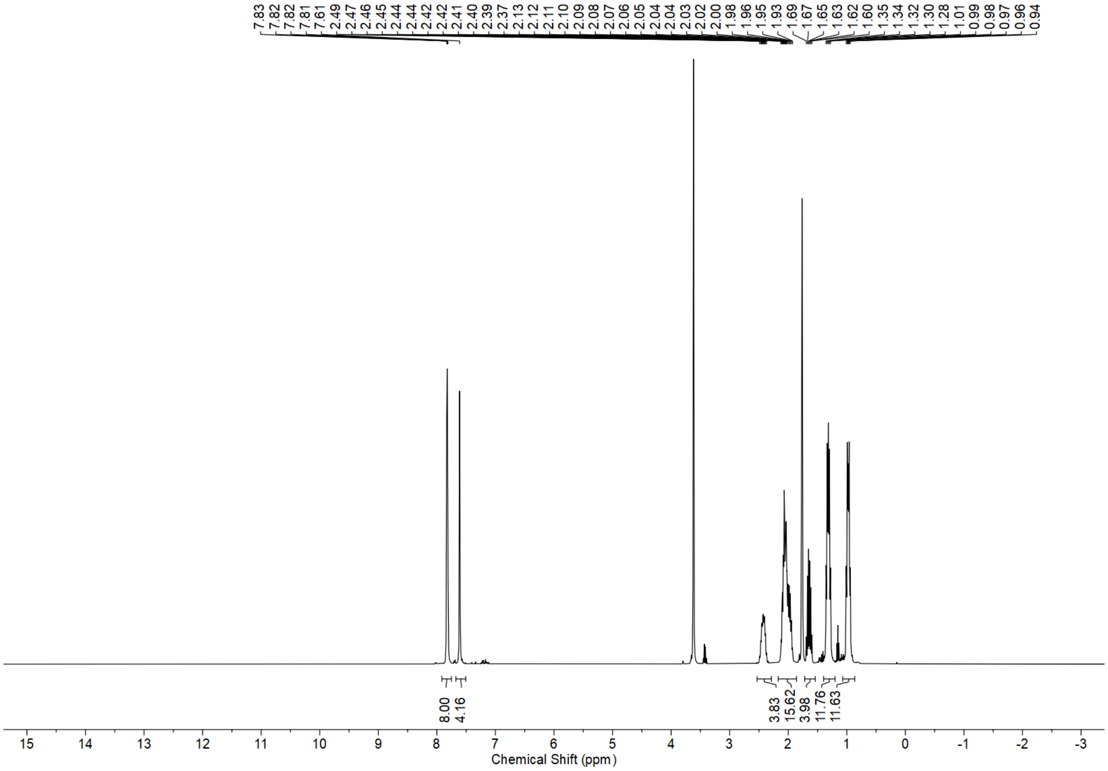

Figure S7. ^{1}H NMR spectrum of **Cr3** in THF-d_8.

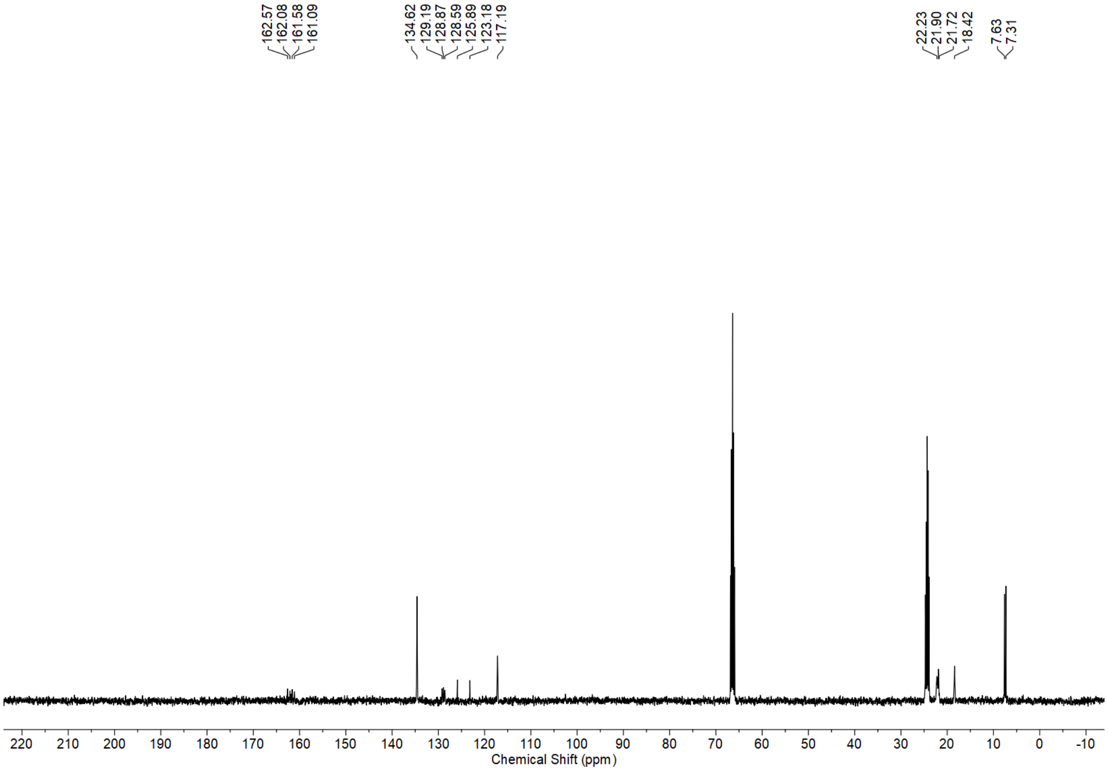

Figure S8. $^{13}C\{^{1}H\}$ NMR spectrum of **Cr3** in THF-d_8.

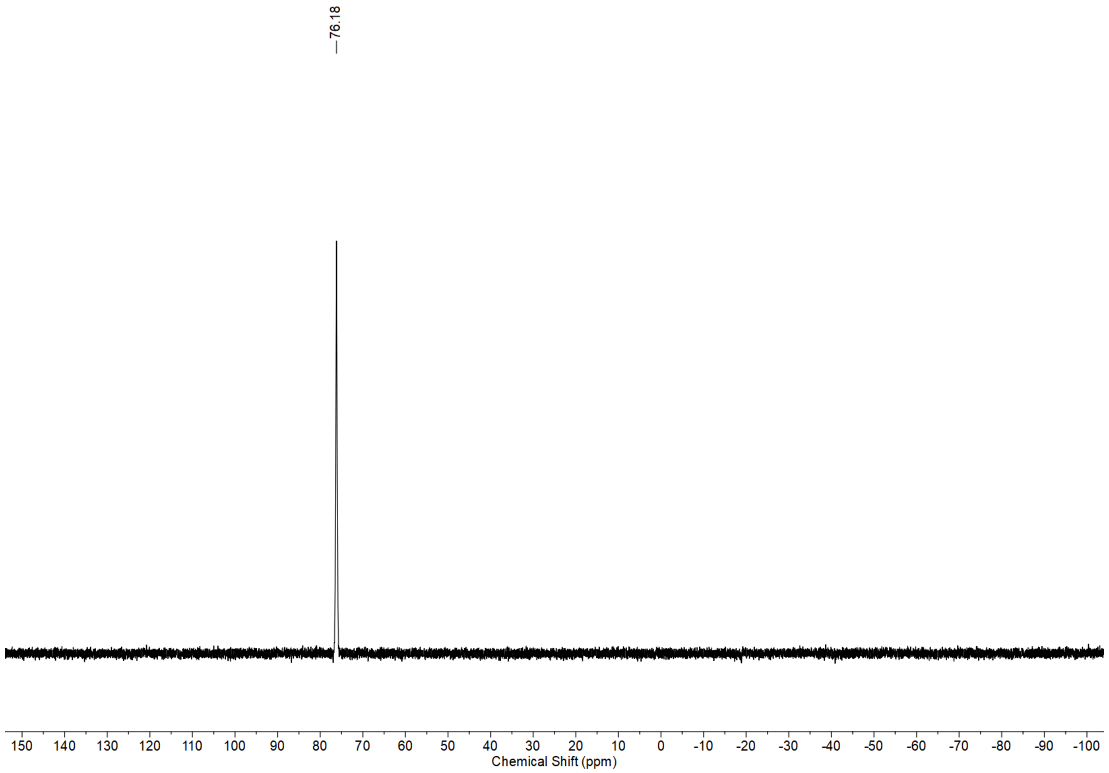

Figure S9. $^{31}P\{^{1}H\}$ NMR spectrum of **Cr3** in THF-d_8.

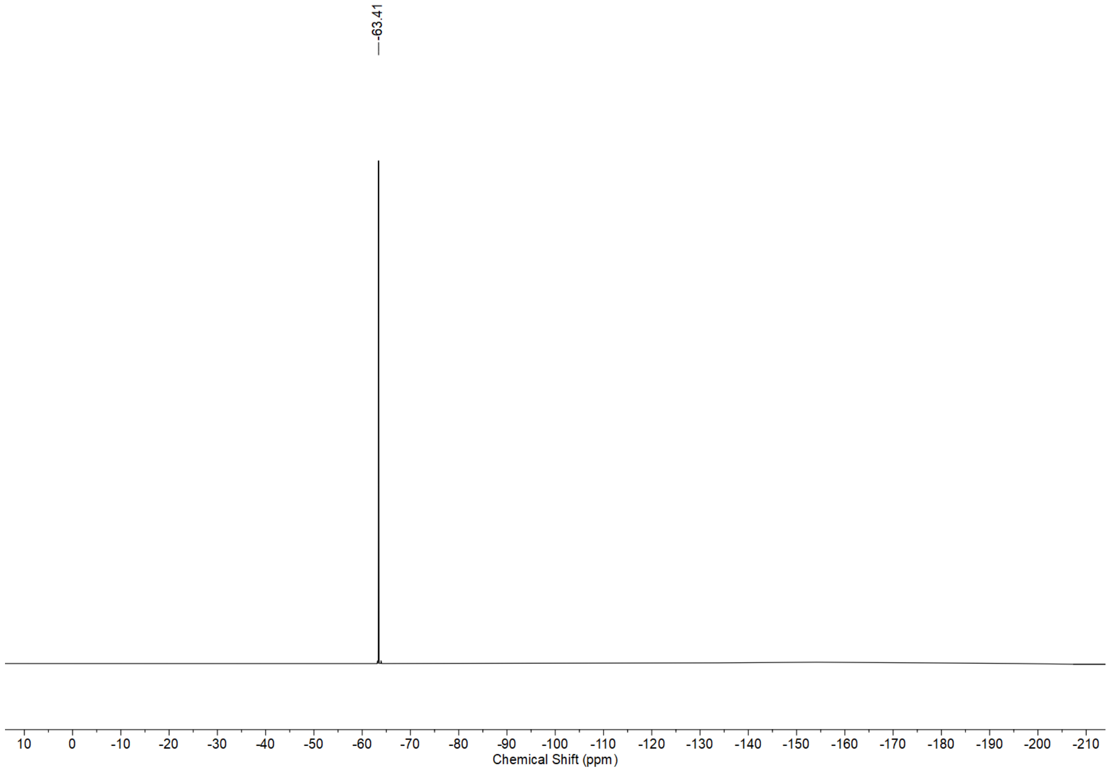

Figure S10. $^{19}F\{^{1}H\}$ NMR spectrum of **Cr3** in THF-d_8.

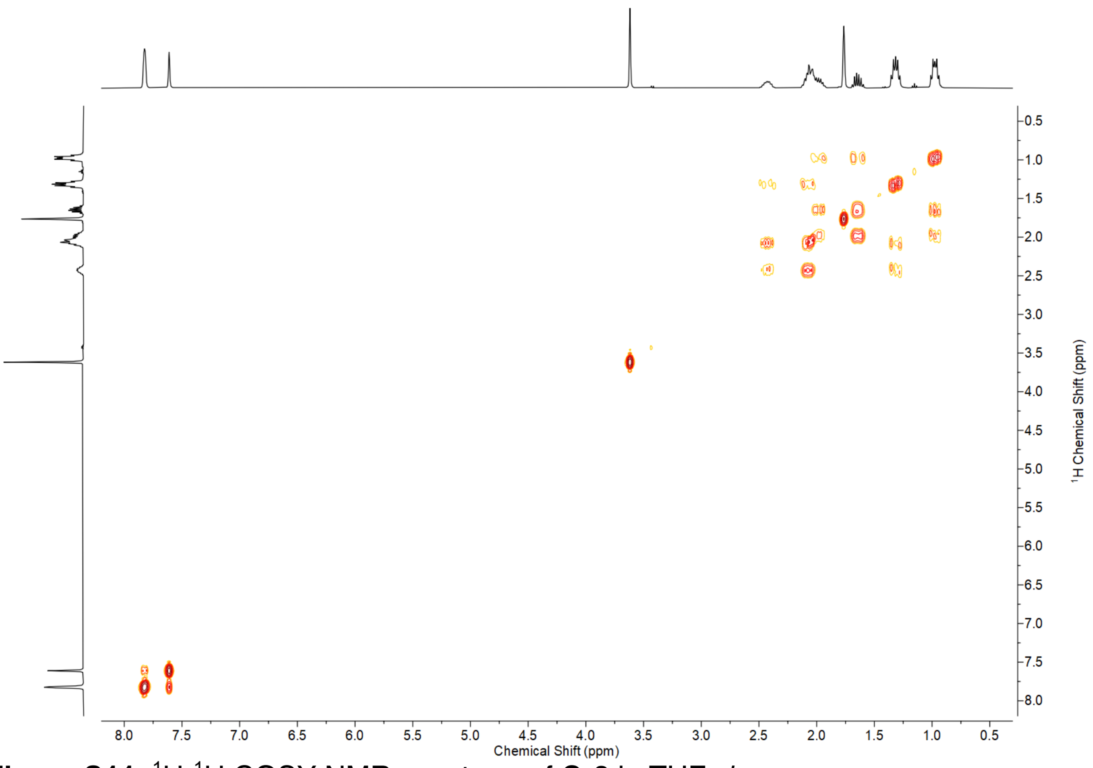

Figure S11. ^{1}H-^{1}H COSY NMR spectrum of **Cr3** in THF-d_8.

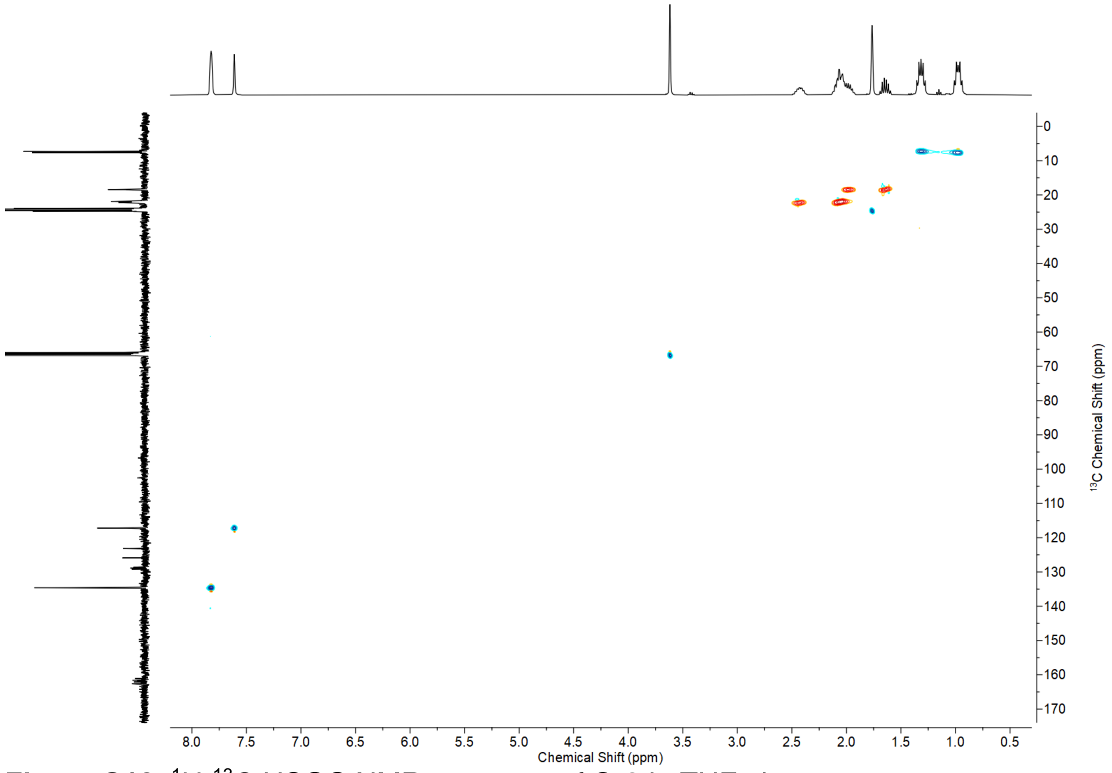

Figure S12. ^{1}H-^{13}C HSQC NMR spectrum of **Cr3** in THF-d_8.

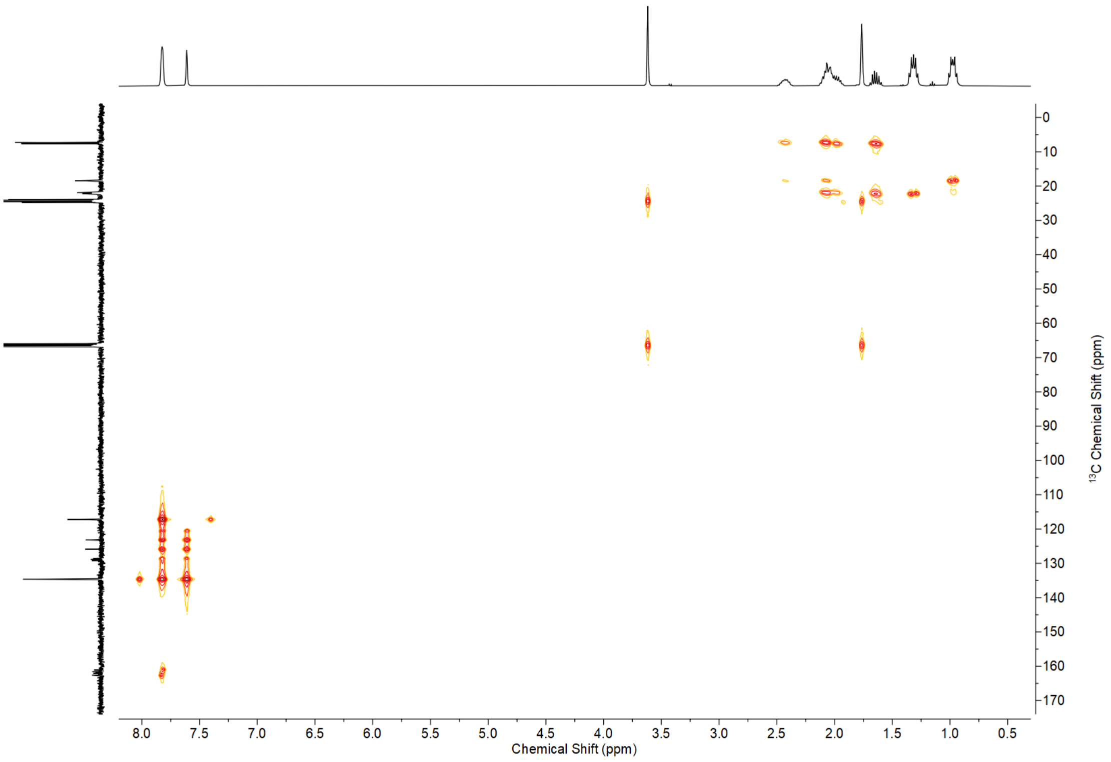

Figure S13. ^{1}H-^{13}C HMBC NMR spectrum of **Cr3** in THF-d_8.

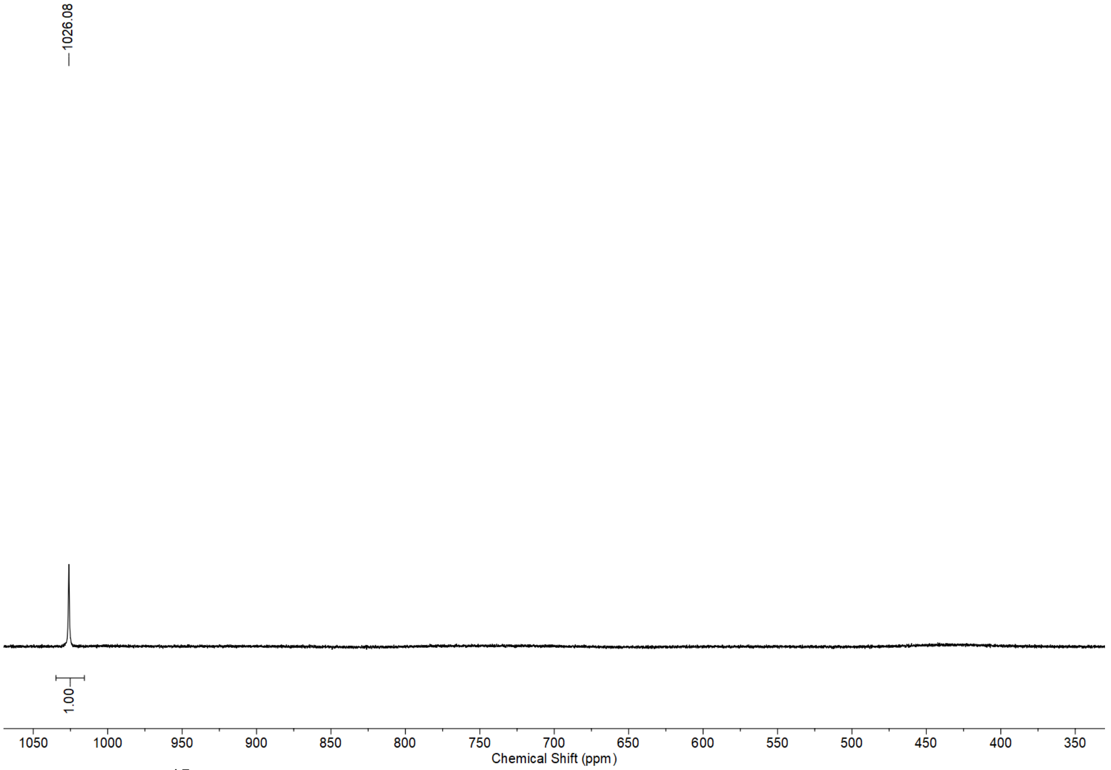

Figure S14. ^{15}N NMR spectrum of **Cr3-^{15}N** in THF-d_8.

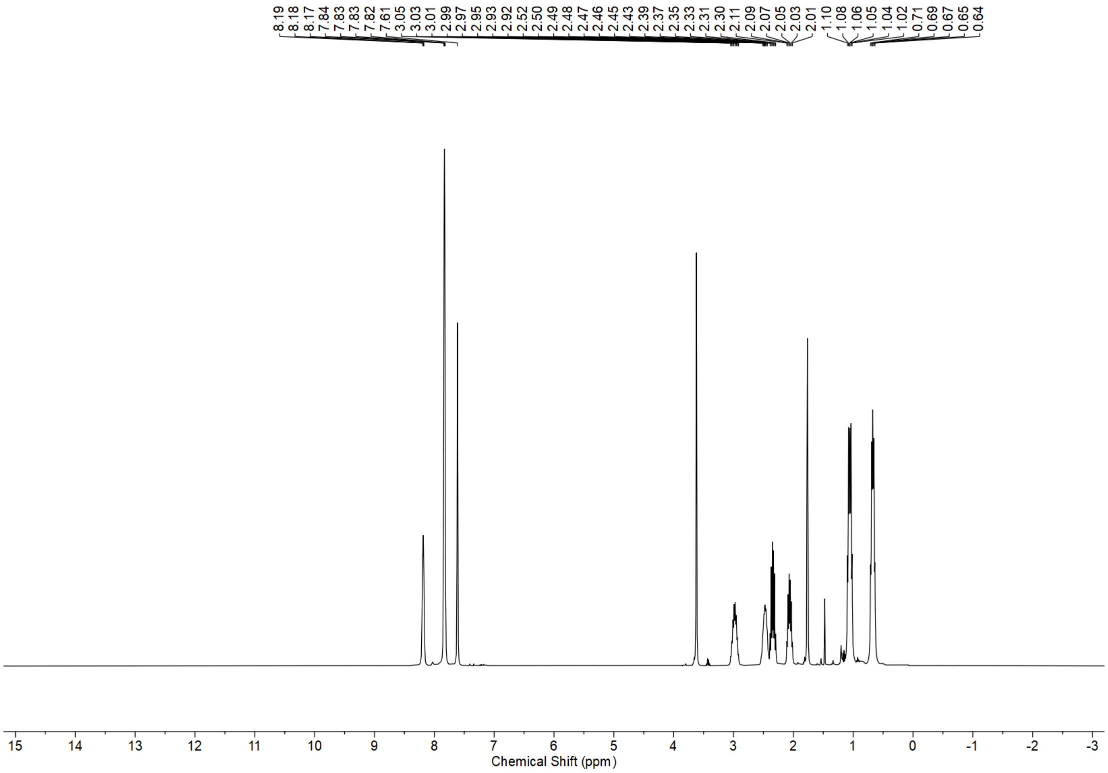

Figure S15. ^{1}H NMR spectrum of **Cr3-benz** in THF-d_8.

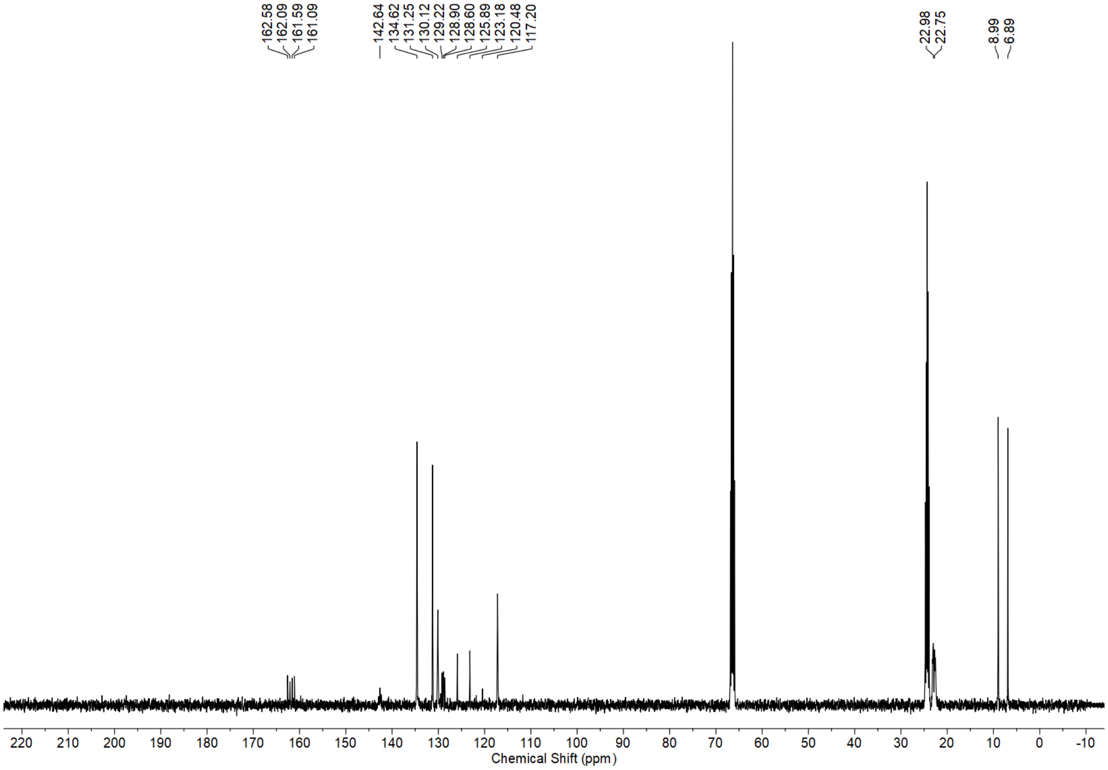

Figure S16. ^{13}C{^{1}H} NMR spectrum of **Cr3** in THF-d_8.

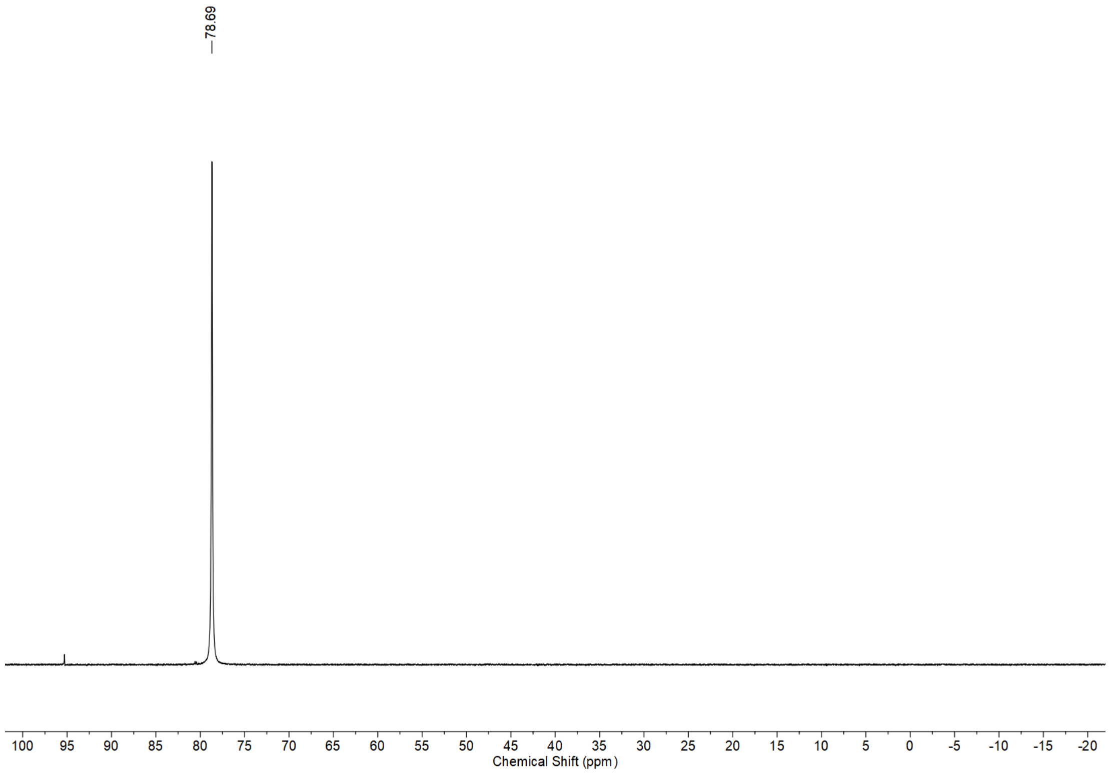

Figure S17. $^{31}P\{^{1}H\}$ NMR spectrum of **Cr3-benz** in THF-d_8.

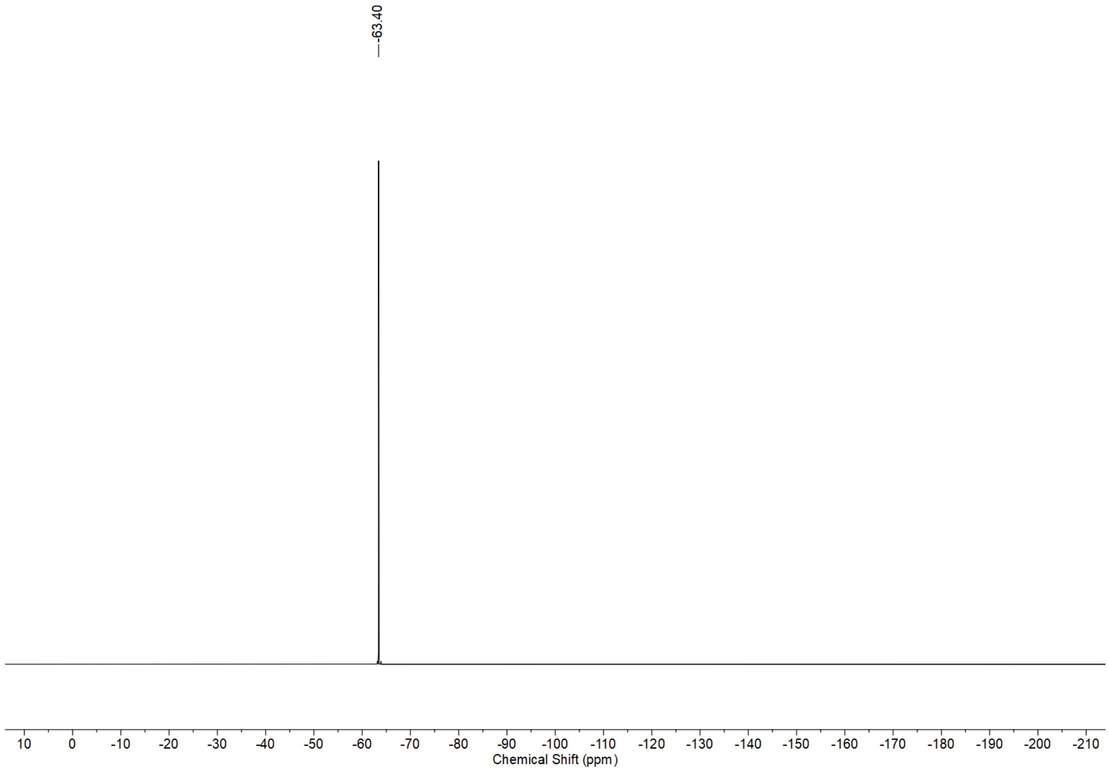

Figure S18. $^{19}F\{^{1}H\}$ NMR spectrum of **Cr3-benz** in THF-d_8.

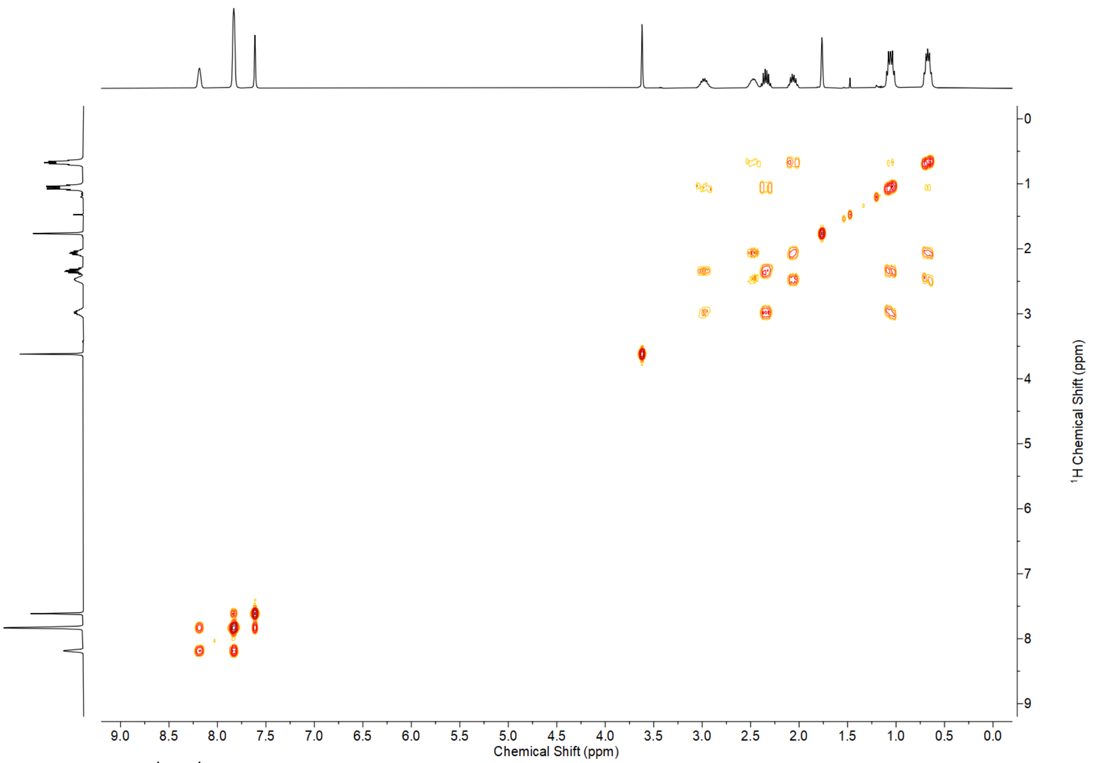

Figure S19. ^{1}H-^{1}H COSY NMR spectrum of **Cr3-benz** in THF-d_8.

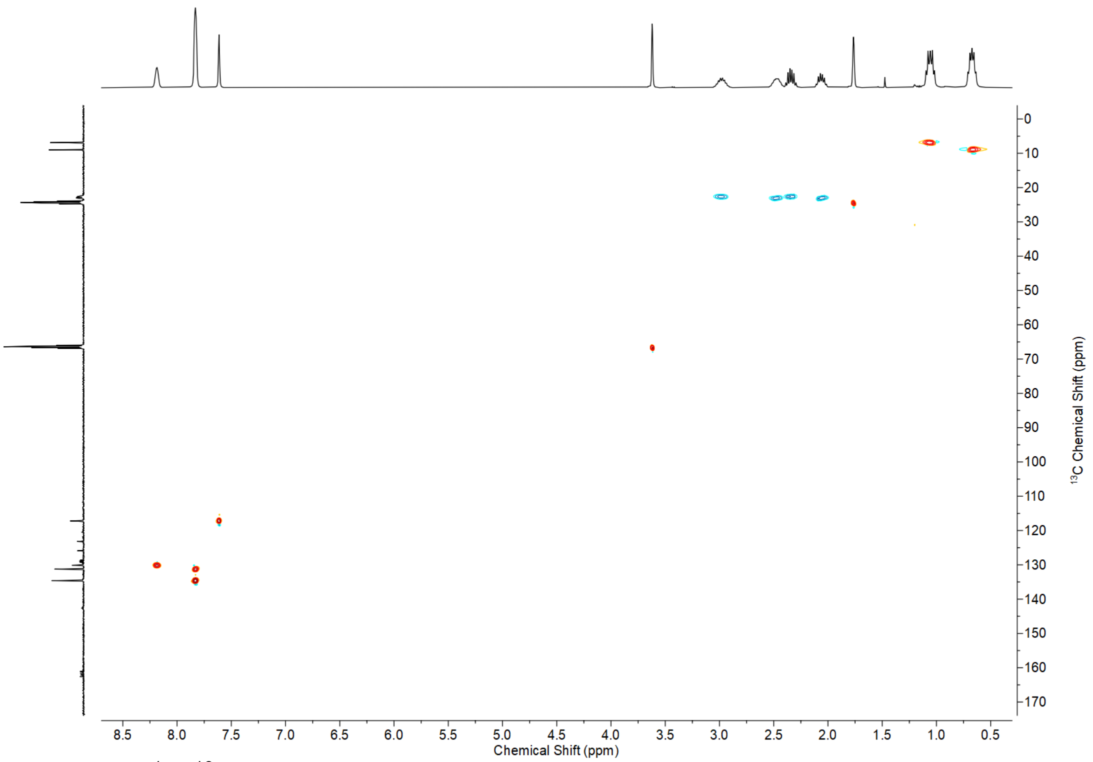

Figure S20. ^{1}H-^{13}C HSQC NMR spectrum of **Cr3-benz** in THF-d_8.

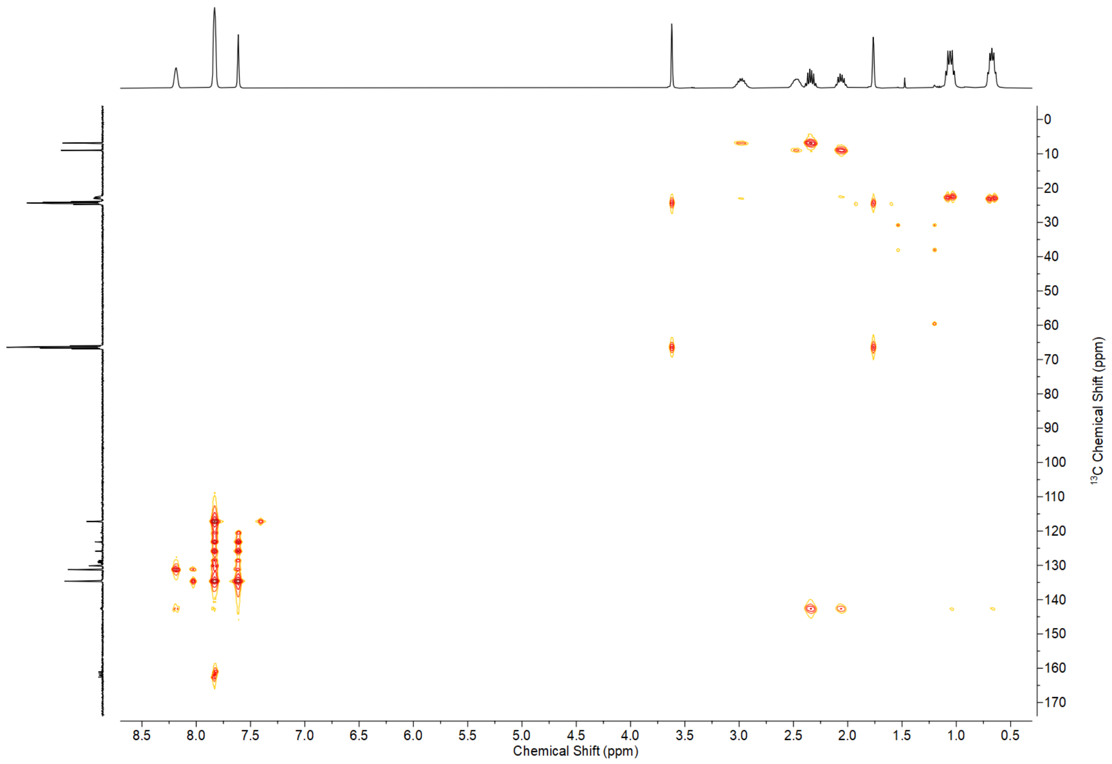

Figure S21. ^{1}H-^{13}C HMBC NMR spectrum of **Cr3-benz** in THF-d_8.

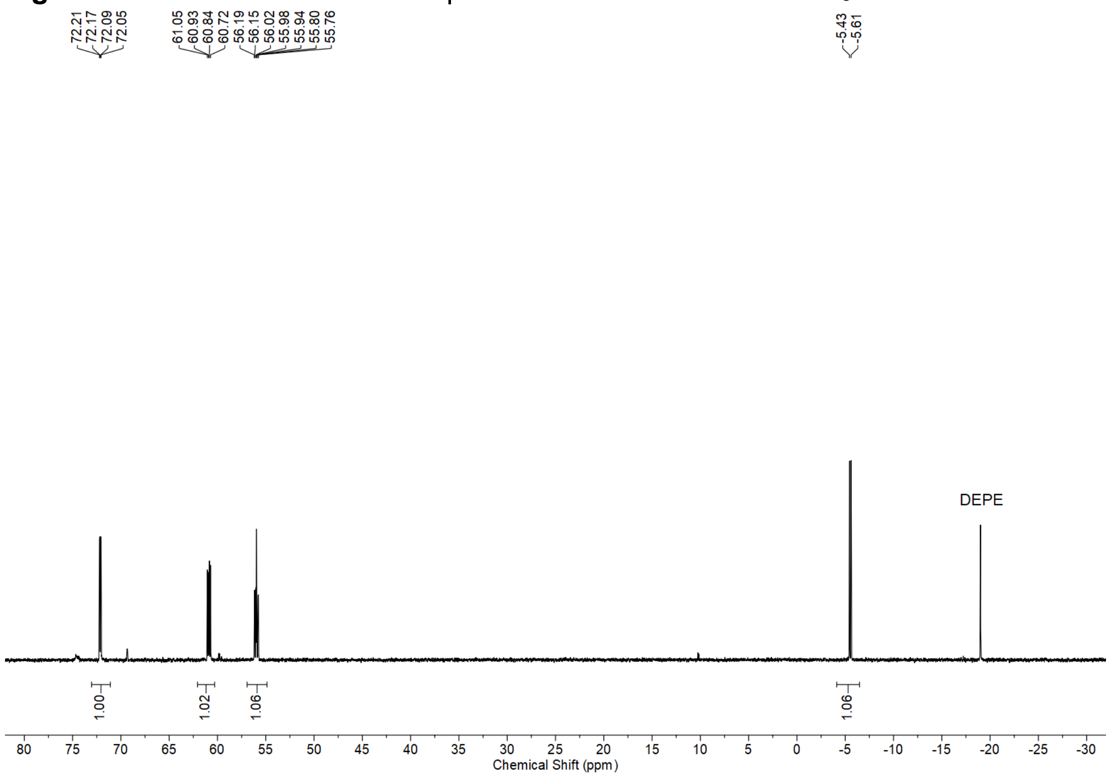

Figure S22. ^{31}P{^{1}H} NMR spectrum of in-situ generated **Cr4** from Cr3 and 2,6-dimethylisocyanide in THF-d_8. Peak at -19.2 ppm is free DEPE ligand.

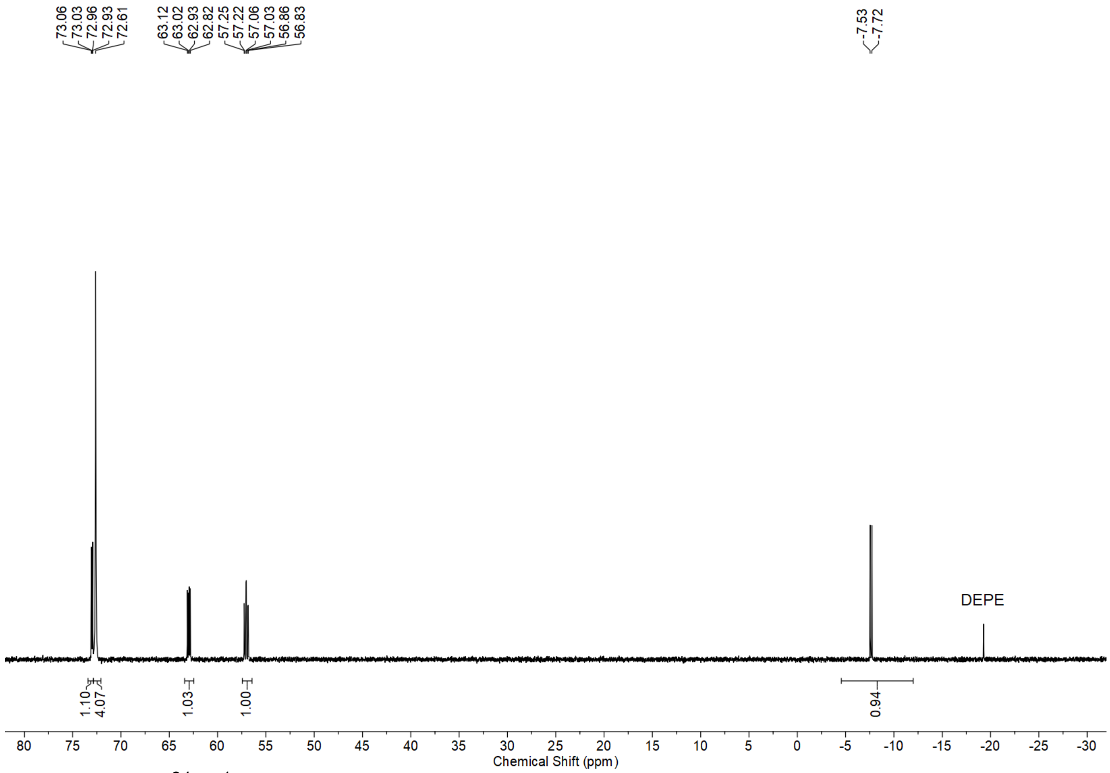

Figure S23. $^{31}P\{^{1}H\}$ NMR spectrum of the reaction of Cr3 and 1-adamantylisocyanide in THF-d_8. Peak at -19.2 ppm is free DEPE ligand.

IV. EPR Spectra

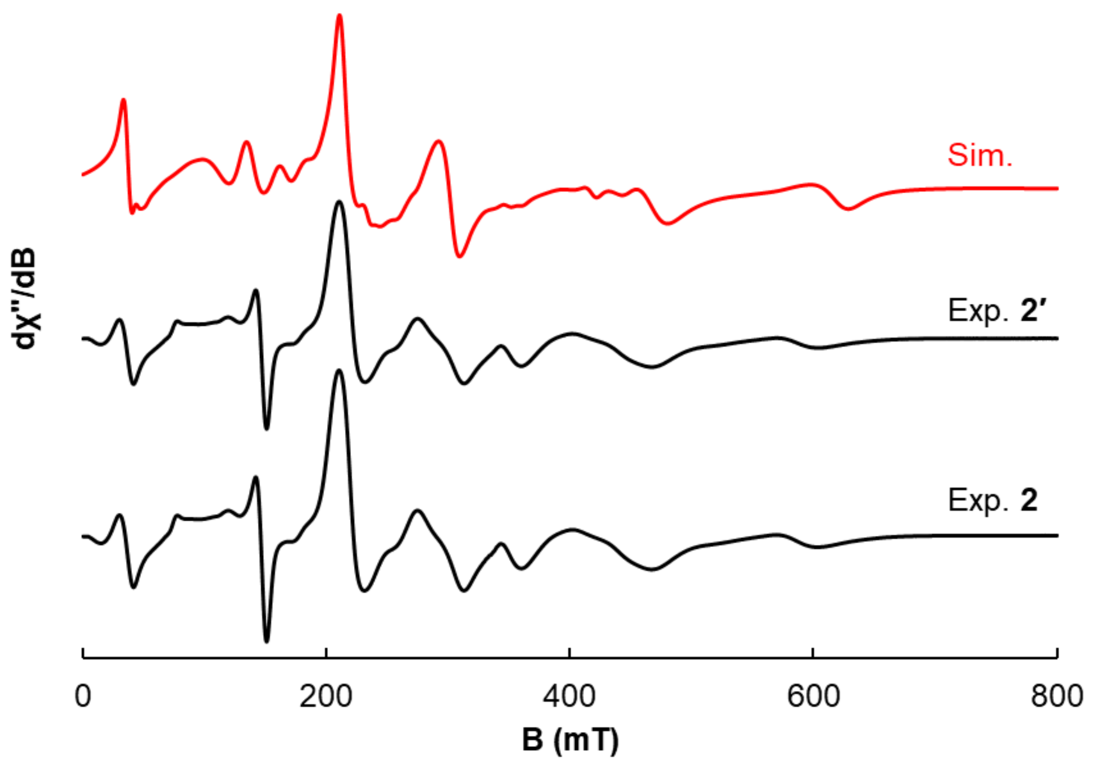

Figure S24. X-Band EPR of **Cr2** spectra collected at 25 K in 2 Me-THF glass. Experimental parameters: microwave frequency = 9.365 GHz, power = 2.000 mW, and modulation amplitude = 4.000 G. Simulation parameters: **Cr2**: S = 5/2, g = 1.943, D = 3495.8, E/D = 0.030, D_{strain} = (103.34, 2149.14)

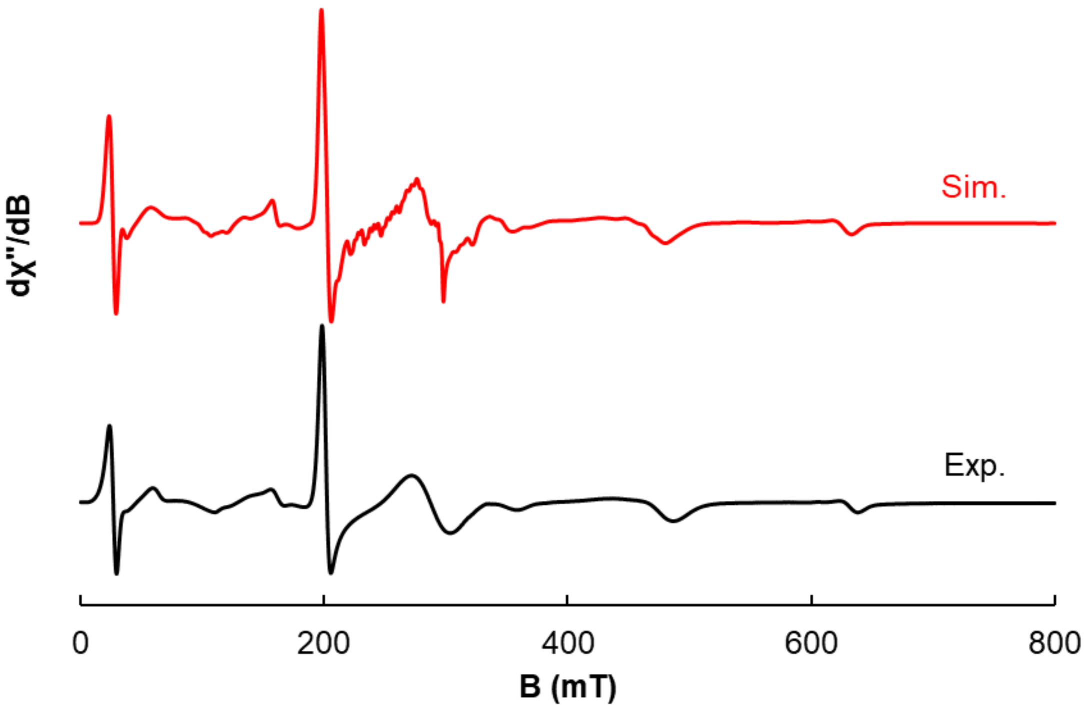

Figure S25. X-Band EPR of **Cr2-benz** spectra collected at 25 K in 2 Me-THF glass. Experimental parameters: microwave frequency = 9.365 GHz, power = 2.000 mW, and modulation amplitude = 4.000 G. Simulation parameters: **Cr2-Benz** S = 5/2, g = 2.001, D = 3798.46, E/D = 0.042, Dstrain = (134.00, 226.92).

V. X-Ray Crystallographic Data

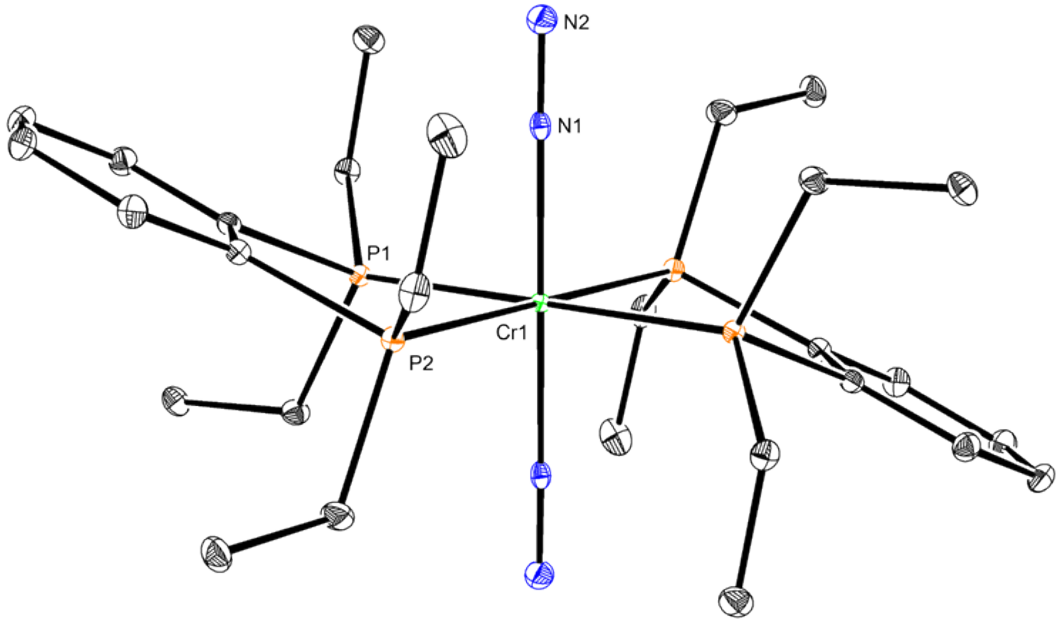

Figure S26. Representation of the solid-state structure of **Cr1-benz** at 30% probability ellipsoids. Hydrogen atoms omitted for clarity.

Table S1. Crystal data for **Cr1-benz**.

Crystal data	
Chemical formula	$2(C_{28}H_{48}CrN_4P_4)$
M_r	1233.16
Crystal system, space group	Monoclinic, $P2_1/n$
Temperature (K)	100
a, *b*, *c* (Å)	10.6428 (2), 13.8840 (2), 10.6636 (2)
b (°)	97.384 (1)
V (Å^3)	1562.64 (5)
Z	1
Radiation type	Cu *K*a
m (mm^{-1})	5.13
Crystal size (mm)	0.37 × 0.25 × 0.11
Data collection	
Diffractometer	Bruker *APEX*-II CCD
Absorption correction	Multi-scan

	SADABS2016/2 (Bruker,2016/2) was used for absorption correction. wR2(int) was 0.0881 before and 0.0376 after correction. The Ratio of minimum to maximum transmission is 0.7831. The I/2 correction factor is Not present.
T_{min}, T_{max}	0.590, 0.753
No. of measured, independent and observed [*I* > 2s(*I*)] reflections	32207, 2774, 2724
R_{int}	0.025
$(\sin q/l)_{max}$ (Å^{-1})	0.596
Refinement	
$R[F^2 > 2s(F^2)]$, $wR(F^2)$, S	0.021, 0.056, 1.06
No. of reflections	2774
No. of parameters	173
H-atom treatment	H-atom parameters constrained
$Dñ_{max}$, $Dñ_{min}$ (e Å^{-3})	0.29, -0.33

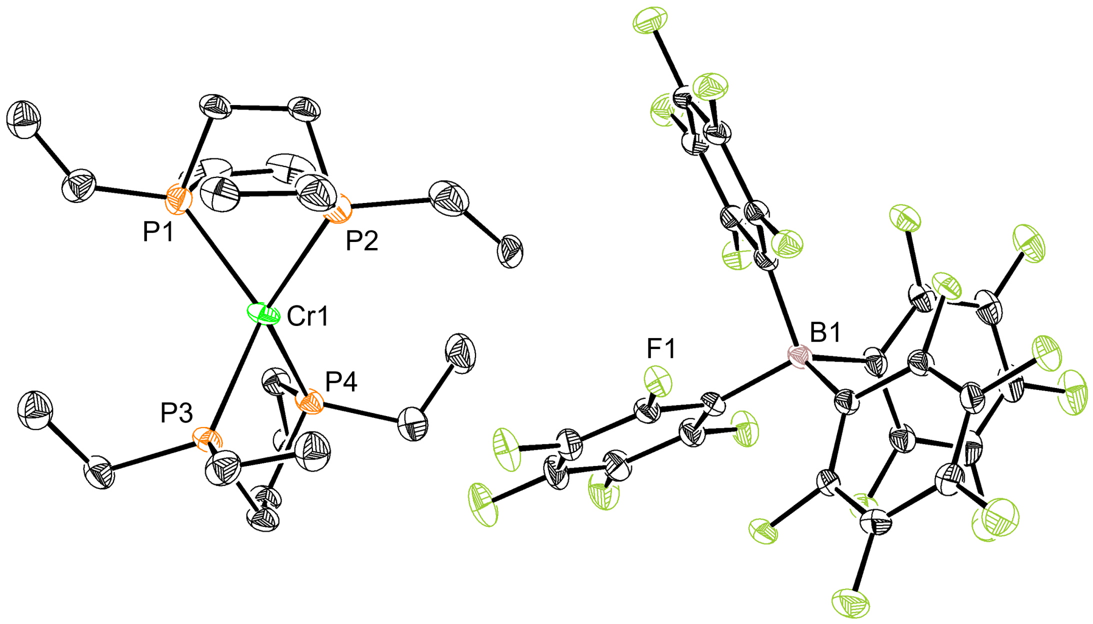

Figure S27. Representation of the solid-state structure of **Cr2'** at 30% probability ellipsoids. Hydrogen atoms and disorder omitted for clarity.

Table S2. Crystal data for **Cr2′**.

Crystal data	
Chemical formula	$C_{20}H_{48}CrP_4{\cdot}BC_{24}F_{20}$
M_r	1143.51
Crystal system, space group	Monoclinic, $P2_1/c$
Temperature (K)	100
a, *b*, *c* (Å)	16.414 (4), 16.789 (6), 18.065 (4)
b (°)	90.79 (2)
V (Å^3)	4978 (2)
Z	4
Radiation type	Cu *K*a
m (mm^{-1})	4.08
Crystal size (mm)	0.41 × 0.19 × 0.16
Data collection	
Diffractometer	Bruker *APEX*-II CCD
Absorption correction	Multi-scan<br>*SADABS2016*/2 (Bruker,2016/2) was

	used for absorption correction. wR2(int) was 0.1015 before and 0.0630 after correction. The Ratio of minimum to maximum transmission is 0.7088. The l/2 correction factor is Not present.
T_{min}, T_{max}	0.534, 0.753
No. of measured, independent and observed [I > 2s(I)] reflections	75877, 8812, 7697
R_{int}	0.049
(sin q/l)$_{max}$ (Å^{-1})	0.596
Refinement	
$R[F^2 > 2s(F^2)]$, $wR(F^2)$, S	0.069, 0.190, 1.06
No. of reflections	8812
No. of parameters	636
No. of restraints	25
H-atom treatment	H-atom parameters constrained
	$w = 1/[s^2(F_o^2) + (0.0921P)^2 + 13.5814P]$ where $P = (F_o^2 + 2F_c^2)/3$
Dñ$_{max}$, Dñ$_{min}$ (e Å^{-3})	1.55, -1.06

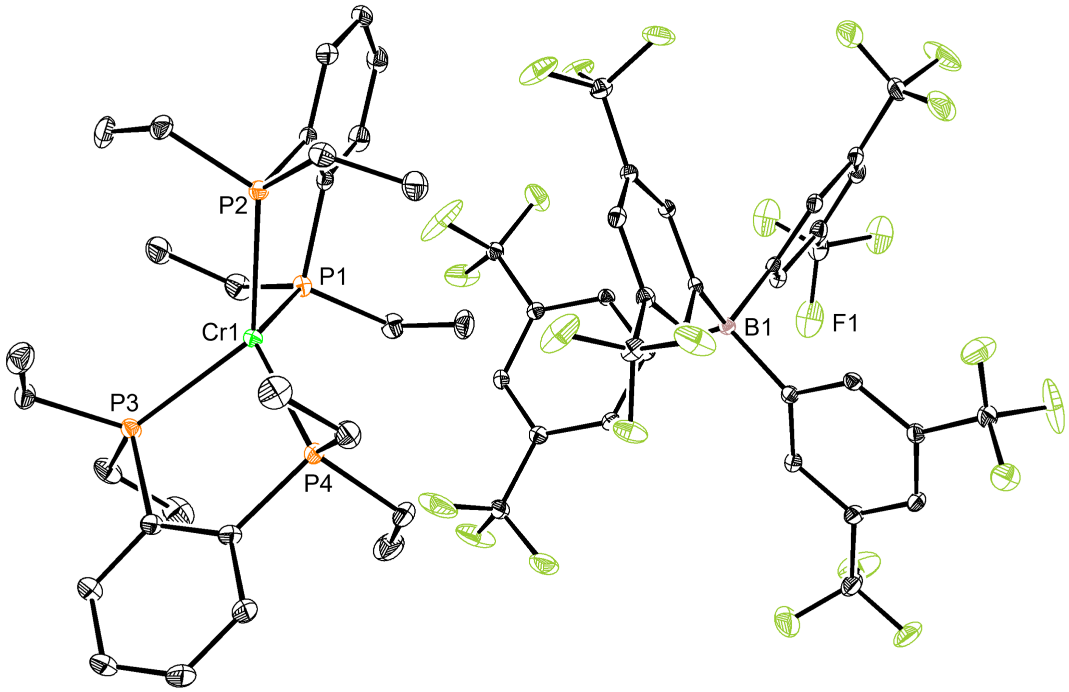

Figure S28. Representation of the solid-state structure of **Cr2-benz** at 30% probability ellipsoids. Hydrogen atoms, equivalent molecule and disorder omitted for clarity.

Table S3. Crystal data for **Cr2-benz**.

Crystal data	
Chemical formula	$C_{28}H_{48}CrP_4{\cdot}C_{32}H_{12}BF_{24}$
*M*r	1423.77
Crystal system, space group	Monoclinic, *P*2$_1$/*n*
Temperature (K)	100
a, *b*, *c* (Å)	12.544 (3), 12.953 (3), 40.032 (12)
b (°)	96.63 (2)
V (Å^3)	6461 (3)
Z	4
Radiation type	Cu *K*a
m (mm^{-1})	3.36
Crystal size (mm)	0.21 × 0.20 × 0.13
Data collection	
Diffractometer	Bruker *APEX*-II CCD
Absorption correction	Multi-scan *SADABS2016*/2 (Bruker,2016/2) was used for absorption correction. wR2(int)

	was 0.1051 before and 0.0475 after correction. The Ratio of minimum to maximum transmission is 0.8304. The l/2 correction factor is Not present.
T_{min}, T_{max}	0.625, 0.753
No. of measured, independent and observed [I > 2s(I)] reflections	73149, 11442, 10283
R_{int}	0.039
(sin q/l)$_{max}$ (Å^{-1})	0.596
Refinement	
$R[F^2 > 2s(F^2)]$, $wR(F^2)$, S	0.049, 0.123, 1.07
No. of reflections	11442
No. of parameters	817
No. of restraints	3
H-atom treatment	H-atom parameters constrained
	$w = 1/[s^2(F_o^2) + (0.0536P)^2 + 11.9261P]$ where $P = (F_o^2 + 2F_c^2)/3$
Dñ$_{max}$, Dñ$_{min}$ (e Å^{-3})	1.38, -1.02

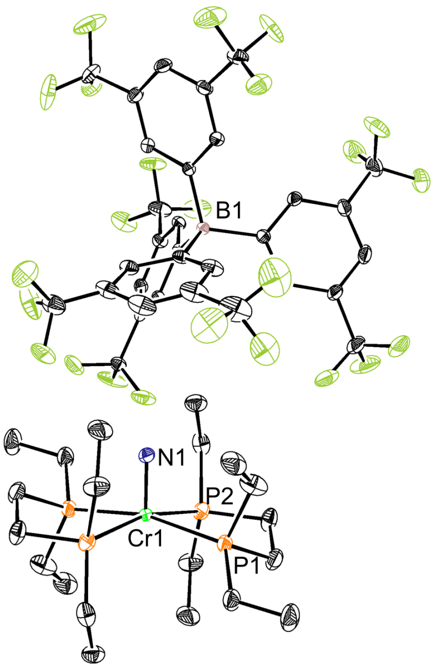

Figure S29. Representation of the solid-state structure of **Cr3** at 30% probability ellipsoids. Hydrogen atoms and disorder omitted for clarity.

Table S4. Crystal data for **Cr3**.

Crystal data	
Chemical formula	$2(C_{10}H_{24}Cr_{0.5}N_{0.5}P_2)\cdot C_{32}H_{12}BF_{24}$
M_r	1341.70
Crystal system, space group	Monoclinic, $P2_1/c$
Temperature (K)	100
a, *b*, *c* (Å)	21.4413 (4), 13.1378 (3), 22.5289 (5)
b (°)	109.661 (1)
V (Å^3)	5976.2 (2)
Z	4
Radiation type	Cu *K*a
m (mm^{-1})	3.59
Crystal size (mm)	0.35 × 0.29 × 0.16
Data collection	

Diffractometer	Bruker *SMART APEX2* area detector
Absorption correction	Multi-scan<br>*SADABS2016*/2 (Bruker,2016/2) was used for absorption correction. wR2(int) was 0.1066 before and 0.0600 after correction. The Ratio of minimum to maximum transmission is 0.7582. The l/2 correction factor is Not present.
T_{min}, T_{max}	0.571, 0.753
No. of measured, independent and observed [I > 2s(I)] reflections	87939, 10962, 9166
R_{int}	0.051
(sin q/l)$_{max}$ (Å^{-1})	0.603
Refinement	
$R[F^2 > 2s(F^2)]$, $wR(F^2)$, S	0.083, 0.214, 1.02
No. of reflections	10962
No. of parameters	1133
No. of restraints	542
H-atom treatment	H-atom parameters constrained
	$w = 1/[s^2(F_o^2) + (0.0855P)^2 + 26.3972P]$ where $P = (F_o^2 + 2F_c^2)/3$
Dρ$_{max}$, Dρ$_{min}$ (e Å^{-3})	1.33, -0.99

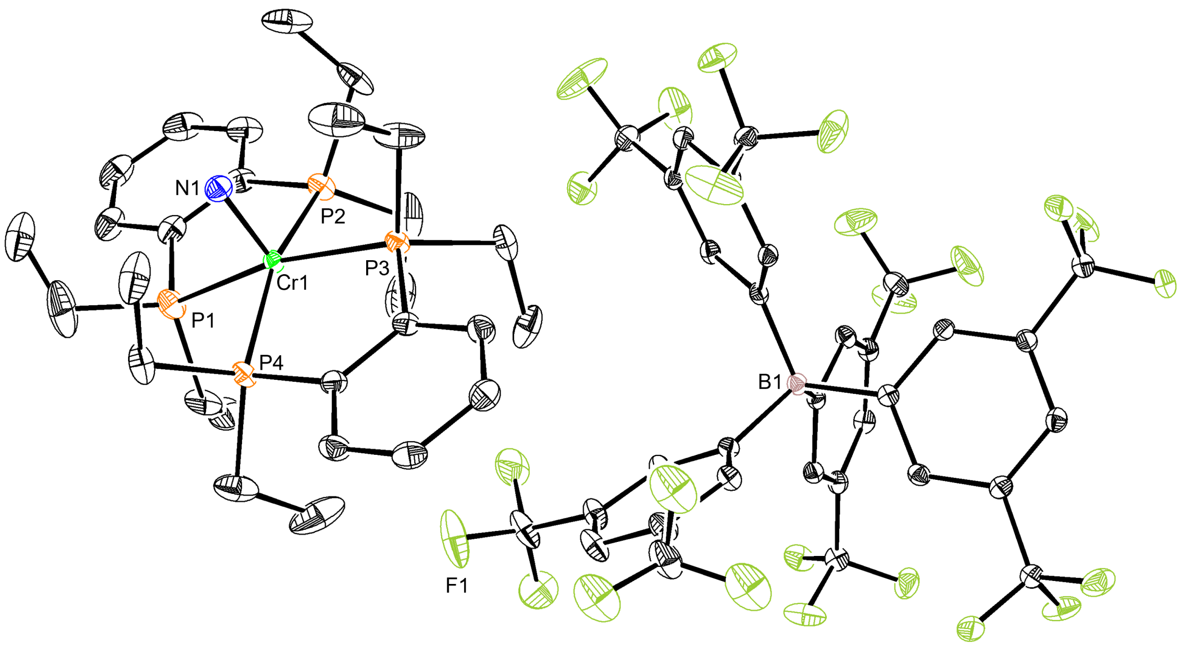

Figure S30. Representation of the solid-state structure of **Cr3-benz** at 30% probability ellipsoids. Hydrogen atoms and disorder omitted for clarity.

Table S5. Crystal data for **Cr3-benz**.

Crystal data	
Chemical formula	$C_{28}H_{44.468}CrNP_4 \cdot C_{32}H_{12}BF_{24}$
M_r	1434.22
Crystal system, space group	Monoclinic, $P2_1/c$
Temperature (K)	100
a, *b*, *c* (Å)	21.3956 (4), 13.0200 (3), 24.9051 (5)
b (°)	112.069 (1)
V (Å^3)	6429.5 (2)
Z	4
Radiation type	Cu *K*a
m (mm^{-1})	3.39
Crystal size (mm)	0.17 × 0.16 × 0.11
Data collection	
Diffractometer	Bruker *APEX*-II CCD
Absorption correction	Multi-scan<br>*SADABS2016*/2 (Bruker,2016/2) was

	used for absorption correction. wR2(int) was 0.0883 before and 0.0646 after correction. The Ratio of minimum to maximum transmission is 0.8766. The l/2 correction factor is Not present.
T_{min}, T_{max}	0.660, 0.753
No. of measured, independent and observed [I > 2s(I)] reflections	132332, 11380, 9501
R_{int}	0.057
(sin q/l)$_{max}$ (Å^{-1})	0.596
Refinement	
$R[F^2 > 2s(F^2)]$, $wR(F^2)$, S	0.063, 0.176, 1.05
No. of reflections	11380
No. of parameters	877
No. of restraints	50
H-atom treatment	H-atom parameters constrained
	$w = 1/[s^2(F_o^2) + (0.0854P)^2 + 16.442P]$ where $P = (F_o^2 + 2F_c^2)/3$
Dñ$_{max}$, Dñ$_{min}$ (e Å^{-3})	1.75, -0.77

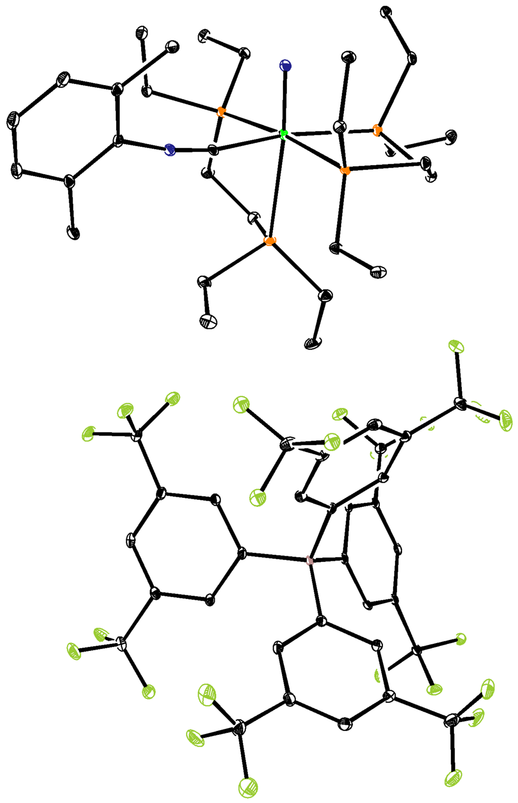

Figure S31. Representation of the solid-state structure of **Cr4** at 30% probability ellipsoids. Hydrogen atoms and disorder omitted for clarity.

Table S6. Crystal data for **Cr4**.

Crystal data	
Chemical formula	$C_{29}H_{57}CrN_2P_4 \cdot C_{32}H_{12}BF_{24}$
M_r	1472.87
Crystal system, space group	Triclinic, $P\bar{1}$
Temperature (K)	100
a, *b*, *c* (Å)	13.4445 (3), 19.6977 (6), 26.7066 (7)
a, b, g (°)	101.341 (2), 92.560 (2), 103.897 (2)
V (Å^3)	6700.1 (3)
Z	4
Radiation type	Cu *K*a
m (mm^{-1})	3.27
Crystal size (mm)	××

Data collection	
Diffractometer	Bruker *APEX*-II CCD
Absorption correction	Multi-scan<br>*SADABS2016*/2 (Bruker,2016/2) was used for absorption correction. wR2(int) was 0.1459 before and 0.0883 after correction. The Ratio of minimum to maximum transmission is 0.6745. The l/2 correction factor is Not present.
T_{min}, T_{max}	0.508, 0.753
No. of measured, independent and observed [I > 2s(I)] reflections	143336, 23479, 17854
R_{int}	0.095
(sin q/l)$_{max}$ (Å^{-1})	0.596
Refinement	
$R[F^2 > 2s(F^2)]$, $wR(F^2)$, S	0.100, 0.290, 1.04
No. of reflections	23479
No. of parameters	1711
No. of restraints	403
H-atom treatment	H-atom parameters constrained
	$w = 1/[s^2(F_o^2) + (0.1598P)^2 + 28.0397P]$ where $P = (F_o^2 + 2F_c^2)/3$
Dρ$_{max}$, Dρ$_{min}$ (e Å^{-3})	1.87, -1.29

VI. Electrochemical Data

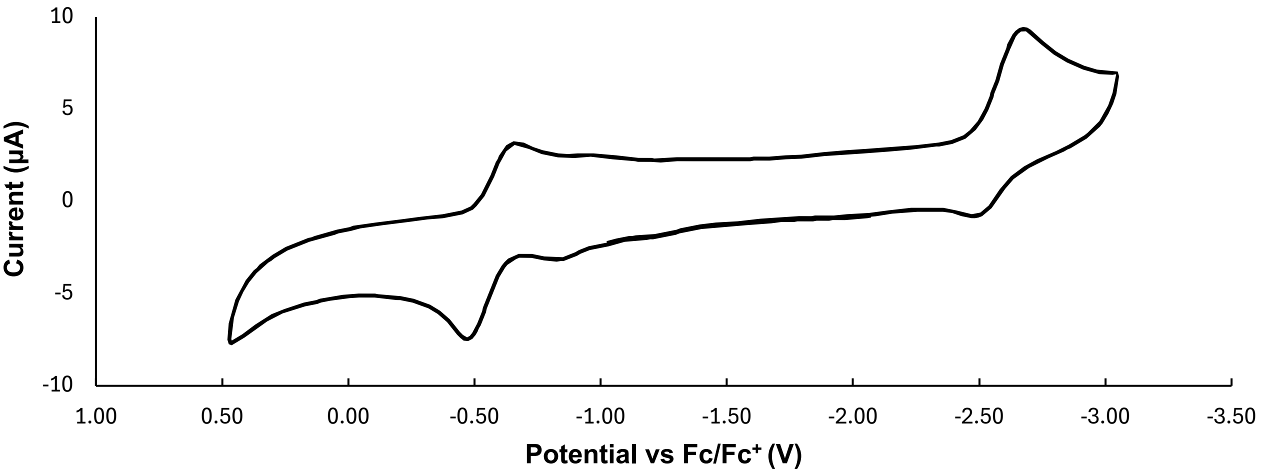

Figure S32. Cyclic voltammogram of 0.75 mM **Cr3** in THF containing 0.1 M $TBAPF_6$ (scan rate 100 mV/s) at 23 °C versus Fc/Fc^+.

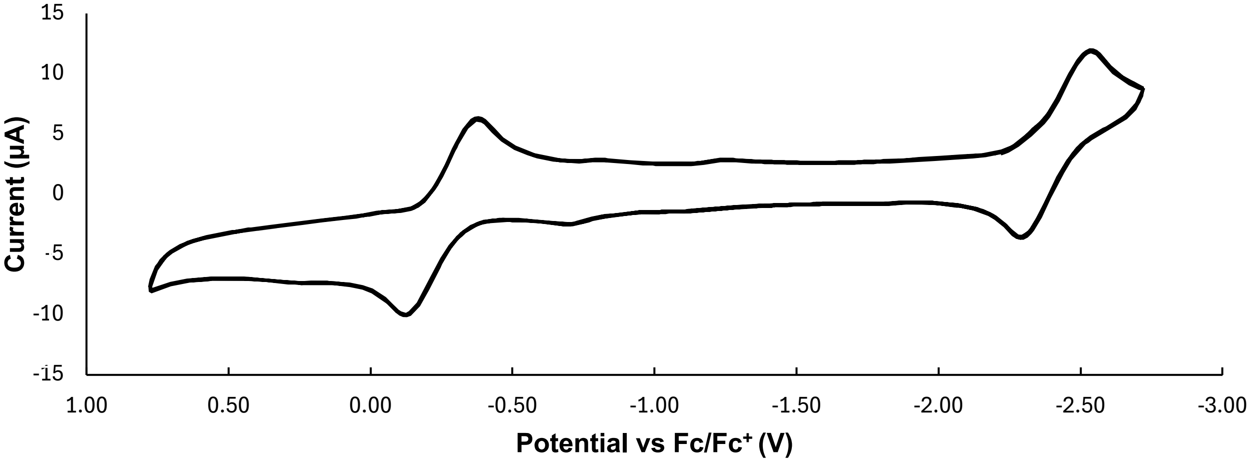

Figure S33. Cyclic voltammogram of 1 mM **Cr3-benz** in THF containing 0.1 M $TBAPF_6$ (scan rate 100 mV/s) at 23 °C versus Fc/Fc^+.

VII. IR Spectra

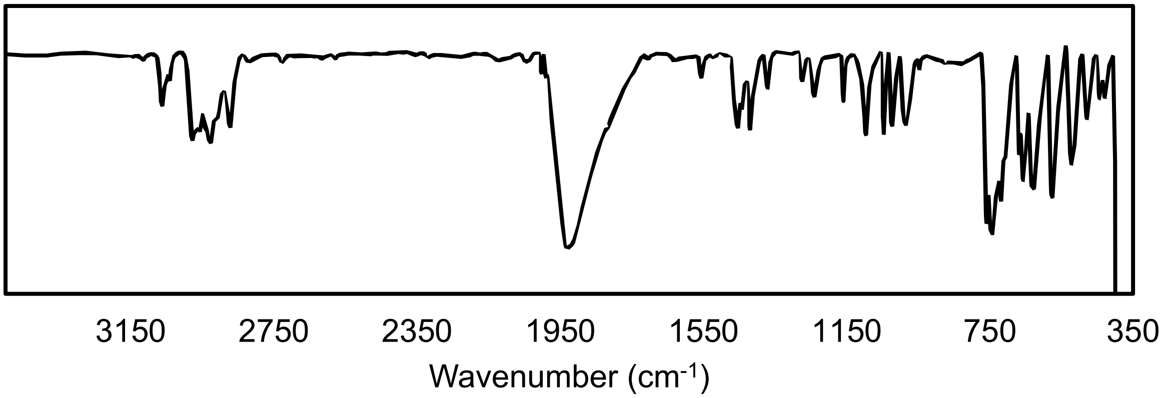

Figure S34. FTIR spectra of **Cr1-benz** in a KBr pellet.

VIII. References

1. Pangborn, A. B.; Giardello, M. A.; Grubbs, R. H.; Rosen, R. K.; Timmers, F. J. Safe and Convenient Procedure for Solvent Purification. *Organometallics* **1996**, *15*, 1518–1520.

2. Inoue, R.; Yamaguchi, M.; Murakami, Y.; Okano, K.; Mori, A. Revisiting of Benzophenone Ketyl Still: Use of a Sodium Dispersion for the Preparation of Anhydrous Solvents. *ACS Omega* **2018**, *3*, 12703–12706.

3. Fulmer, G. R.; Miller, A. J. M.; Sherden, N. H.; Gottlieb, H. E.; Nudelman, A.; Stoltz, B. M.; Bercaw, J. E.; Goldberg, K. I. NMR Chemical Shifts of Trace Impurities: Common Laboratory Solvents, Organics, and Gases in Deuterated Solvents Relevant to the Organometallic Chemist. *Organometallics* **2010**, *29*, 2176–2179.

4. Stoll, S.; Schweiger, A. EasySpin, a comprehensive software package for spectral simulation and analysis in EPR. *J. Magn. Reson.* **2006**, *178*, 42–55.

5. Günther, H.; Tambornino, F. Trityl isocyanate, isothiosyanate and azide- Syntheses, crystallographic studies and Hirshfeld surface analysis. *J. Mol. Struc.* **2025**, *1332*, 141636.

6. I. Chávez, A, Alvarez-Carena, E. Molins, A. Roig, W. Maniukiewicz, A. Arancibia, V. Arancibia, H. Brand, J. M. Manrıquez, Selective oxidants for organometallic compounds containing a stabilising anion of highly reactive cations: $(3,5(CF_3)_2C_6H_3)_4B^-)Cp_2Fe^+$ and $(3,5(CF_3)_2C_6H_3)_4B^-)Cp^*{}_2Fe^+$. *J. Organomet. Chem.* **2000**, *601*, 126–132.

7. Ehudin, M. A.; Gee, L. B.; Sabuncu, S.; Braun, A.; Moënne-Loccoz, P.; Hedman, B.; Hodgson, K. O.; Solomon, E. I.; Karlin, K. D. Tuning the Geometric and Electronic Structure of Synthetic High-Valent Heme Iron(IV)-Oxo Models in the Presence of a Lewis Acid and Various Axial Ligands. *J. Am. Chem. Soc.* **2019**, *141*, 5942–5960.

8. Ricci, G.; Forni, A.; Boglia, A.; Sonzogni, M. New Chromium(II) Bidentate Phosphine Complexes: Synthesis, Characterization, and Behavior in the Polymerization of 1,3-Butadiene. *Organometallics.* **2004**, *23*, 3727-3732.

9. Beasley, C. H.; Duletski, O. L.; Stankevich, K. S.; Arulsamy, N.; Mock, M. T. Catalytic dinitrogen reduction to hydrazine and ammonia using $Cr(N_2)_2(diphosphine)_2$ complexes. *Dalton Trans.* **2024**, *53*, 6496-6500.
